# The histone methyltransferase SETD2 regulates HIV expression and latency

**DOI:** 10.1371/journal.ppat.1012281

**Published:** 2024-06-07

**Authors:** Cameron R. Bussey-Sutton, Airlie Ward, Joshua A. Fox, Anne-Marie W. Turner, Jackson J. Peterson, Ann Emery, Arturo R. Longoria, Ismael Gomez-Martinez, Corbin Jones, Austin Hepperla, David M. Margolis, Brian D. Strahl, Edward P. Browne

**Affiliations:** 1 Department of Biochemistry, UNC Chapel Hill, Chapel Hill, North Carolina, United States of America; 2 Department of Medicine, UNC Chapel Hill, Chapel Hill, North Carolina, United States of America; 3 UNC HIV Cure Center, UNC Chapel Hill, Chapel Hill, North Carolina, United States of America; 4 Department of Microbiology and Immunology, UNC Chapel Hill, Chapel Hill, North Carolina, United States of America; 5 Lineberger Comprehensive Cancer Center, UNC Chapel Hill, Chapel Hill, North Carolina, United States of America; 6 Department of Genetics, UNC Chapel Hill, Chapel Hill, North Carolina, United States of America; Institut Cochin, INSERM U1016, FRANCE

## Abstract

Understanding the mechanisms that drive HIV expression and latency is a key goal for achieving an HIV cure. Here we investigate the role of the SETD2 histone methyltransferase, which deposits H3K36 trimethylation (H3K36me3), in HIV infection. We show that prevention of H3K36me3 by a potent and selective inhibitor of SETD2 (EPZ-719) leads to reduced post-integration viral gene expression and accelerated emergence of latently infected cells. CRISPR/Cas9-mediated knockout of SETD2 in primary CD4 T cells confirmed the role of SETD2 in HIV expression. Transcriptomic profiling of EPZ-719-exposed HIV-infected cells identified numerous pathways impacted by EPZ-719. Notably, depletion of H3K36me3 prior to infection did not prevent HIV integration but resulted in a shift of integration sites from highly transcribed genes to quiescent chromatin regions and to polycomb repressed regions. We also observed that SETD2 inhibition did not apparently affect HIV RNA levels, indicating a post-transcriptional mechanism affecting HIV expression. Viral RNA splicing was modestly reduced in the presence of EPZ-719. Intriguingly, EPZ-719 exposure enhanced responsiveness of latent HIV to the HDAC inhibitor vorinostat, suggesting that H3K36me3 can contribute to a repressive chromatin state at the HIV locus. These results identify SETD2 and H3K36me3 as novel regulators of HIV integration, expression and latency.

## Author summary

HIV persists in people during therapy by entering a state of latency, where viral genes are not expressed. This reservoir of latently infected cells is the key barrier to curing HIV. In this manuscript, we examine the role of the cellular enzyme SETD2 in HIV infection and expression. SETD2 generates H3K36me3 modifications on histone proteins that associate with cellular DNA. We find that H3K36me3 modifications are abundantly present on the integrated HIV genome during infection. We observed that inhibiting SETD2 with the compound EPZ-719 leads to HIV integrating into different regions of the human genome and reduces HIV expression. Interestingly, this also increased the sensitivity of latent HIV to reactivation by histone deacetylase inhibitors. EPZ-719 did not apparently impact the transcription of HIV RNA, but affected splicing of the viral RNA into coding transcripts. Overall, these results show that SETD2 influences HIV integration and expression. SETD2 may be a useful new target for reactivating HIV gene expression in people with HIV.

## Introduction

Antiretroviral therapy (ART) targeting key HIV enzymes has been enormously successful at treating HIV infection and has allowed people with HIV (PWH) to lead relatively normal lives. Nevertheless, a cure for HIV has remained elusive, and interruption of ART results in rapid viral rebound [1,2]. The details of how HIV is able to persist during decades of therapy are not entirely clear, but likely involve the ability of HIV to enter a state of latent infection in memory CD4 T cells. In these cells, HIV expression is silent or reduced, and latently infected cells are thus able to remain undetected by the immune system. Additionally, the pool of latently infected cells can be replenished by clonal expansion in response to antigenic stimulation or homeostatic cues [3–5].

A key mystery in the field is defining the molecular mechanisms that regulate HIV expression and identifying pathways that contribute to maintaining the latent state. The long-lived HIV reservoir is primarily found in resting CD4 T cells, and these cells exhibit low levels of key activating transcription factors (TFs) such as NF-κB, AP-1 and the elongation promoting complex P-TEFb [6–8]. Furthermore, HIV expression in latently infected cells is repressed by the formation of heterochromatin at the integrated provirus, involving a specific set of covalent histone modifications [8–15]. HIV latency is a stable and heritable phenomenon, consistent with the notion of an epigenetic program that regulates HIV latency [16]. In particular, removal of activating marks such as H3K9ac and H3K27ac by class 1 histone deacetylases (HDACs) and the addition of repressive H3K9me3 and H3K27me3 marks by histone methyltransferases have been shown to contribute to HIV latency [17–19]. ATACseq analysis has also shown that latent proviruses exhibit a “closed” chromatin conformation that likely restricts access by key TFs and RNA Polymerase II (RNAPol2) [16].

Latency reversing agents (LRAs) have been developed that target various HIV regulating transcriptional pathways, including HDAC inhibitors (HDACis), PKC agonists and non-canonical NF-κB agonists [20]. Some compounds have shown promise for reactivating latent HIV in animal models and in clinical studies [18,21–24]. However, no intervention in PWH has yet demonstrated potency for reducing reservoir size. A key issue is that most LRAs are able to reactivate only a fraction of the replication competent reservoir, suggesting that latency is inefficient with single agents [25]. This observation can be explained by a model in which latency is considered as a complex set of states regulated by multiple overlapping mechanisms, and that targeting of multiple pathways concurrently will likely be required to broadly reactivate the reservoir. Thus, fully defining the set of host pathways that impact HIV expression and the key spectrum of limiting pathways in resting CD4 T cells will be crucial for the improvement of latency reversal strategies.

SETD2 is a large (~290kDa) histone methyltransferase that is crucial for the generation of the H3K36me3 mark in metazoan cells [26]. This enzyme is recruited, in part, by phosphorylated serine-2 on the C-terminal domain of RNA Pol 2 to sites of active transcription and co-transcriptionally deposits H3K36me3 marks in gene bodies [27]. The precise molecular function of this histone mark in gene expression has been the subject of considerable investigation. Current data indicates that H3K36me3 helps to prevent cryptic transcriptional initiation within genes [28], as well as to inhibit repressive histone methyltransferases such as the polycomb repressive complex 2 (PRC2) that generates the transcription-inhibiting H3K27me3 mark [29]. H3K36me3 also regulates DNA repair and transcript splicing [30,31]. However, the role of SETD2 and H3K36me3 in HIV infection is unknown. Significantly, HIV integrase interacts with the PWWP domain containing protein LEDGF, a reader of the H3K36me3 mark, raising the possibility that H3K36me3 could potentially contribute to HIV integration [32]. Indeed, in vitro integration assays demonstrate that H3K36me3-modified histones significantly enhance integration of HIV pre-integration complexes (PICs) into nucleosomal substrates [33,34]. Interestingly, it has also recently been shown that the chromatin insulator CTCF also can mediate HIV integration in microglia and is enriched near regions of high H3K36me3 [35]. Given this connection, we sought to understand whether SETD2 and/or H3K36me3 contributes to HIV infection by selective inhibition of the enzyme or by CRISPR/Cas9 directed gene knockout. Our results demonstrate that SETD2 or H3K36me3 is not strictly required for HIV infection but influences the location of integration sites and plays a key role in regulating post-integration HIV expression and latency.

## Results

### SETD2 activity is not required for HIV infection but regulates HIV expression and latency

To analyze the role of SETD2, we employed the highly potent and selective SETD2 inhibitor EPZ-719 to examine the impact of H3K36me3 loss on HIV infection [36]. We initiated our studies in an HIV infected human Jurkat-derived CD4 T cell line (2D10 cells) and observed that exposure of EPZ-719 led to a dose dependent reduction in the level of H3K36me3, with a clear reduction observed by western blot at 100 nM, and a further reduction observable up to 500 nM for a 72h exposure (**S1 Fig, panel A**). We also examined the kinetics of H3K36me3 loss in 2D10 cells by taking daily protein samples after 500 nM EPZ-719 exposure. We observed progressive depletion of H3K36me3 over time in the presence of EPZ-719, with the majority of this mark being lost by 24h, and near complete depletion by 72h (**S1 Fig, panel B**). These observations confirm that 500 nM exposure of 2D10 cells to EPZ-719 is sufficient to lead to potent reduction of H3K36me3 levels in this cell line.

**Fig 1:**
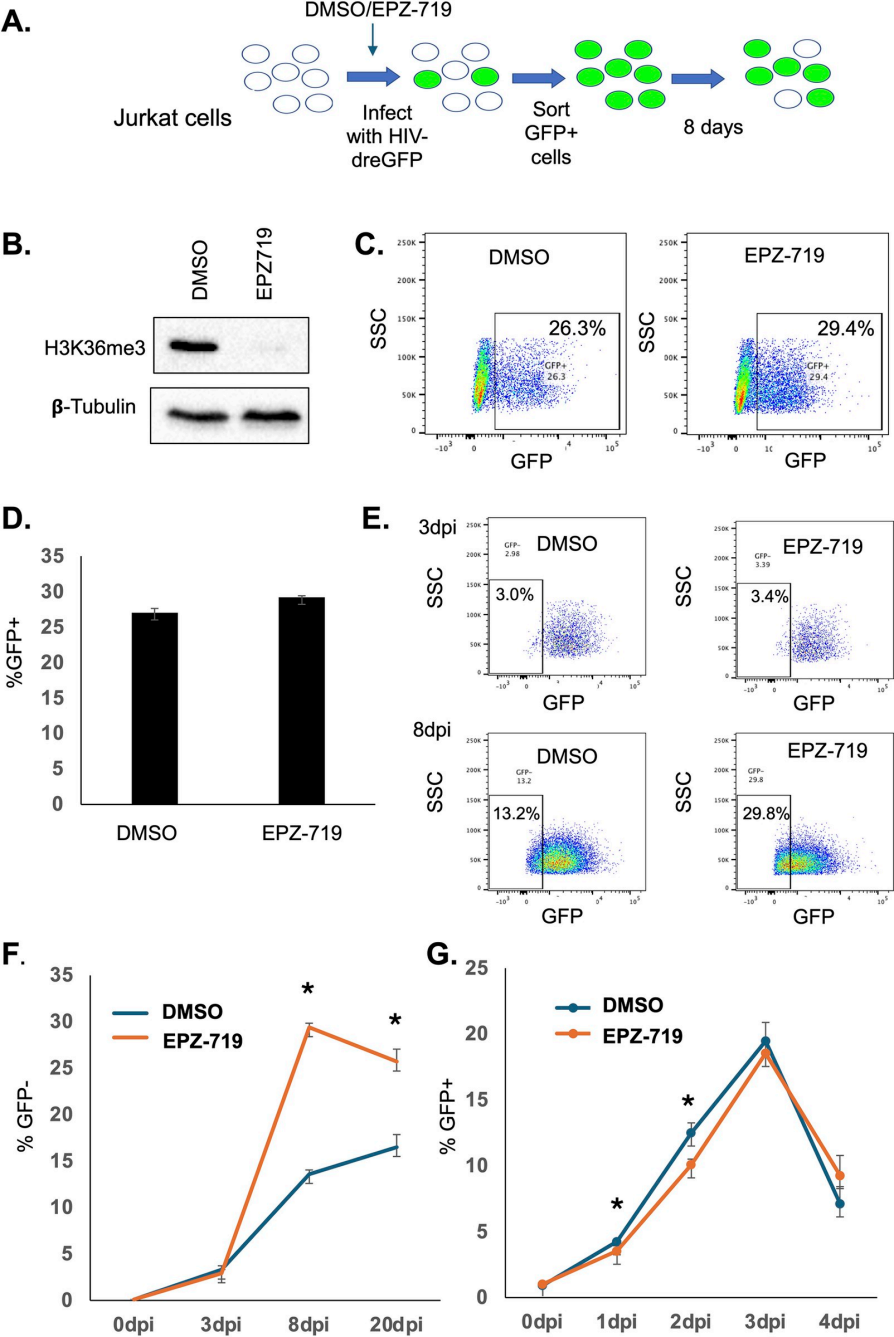
SETD2 activity is not required for HIV infection but regulates HIV expression. (**A**.) Schematic of experimental design for panels B-F. (**B**). Jurkat cells were exposed to EPZ-719 at 500nM for 3 days, then total cell protein lysate was western blotted for H3K36me3 and β-tubulin. (**C**). EPZ-719 treated or control (DMSO) exposed cells (3 days) were infected with HIV-drEGFP, and infection measured at 2dpi by flow cytometry. (**D).** Bar chart of HIV infection for EPZ-719 or DMSO exposed cells. (**E, F**). HIV-dreGFP infected cells were enriched for productively infected cells by flow sporting at 3dpi, then cultured in the presence of EPZ-719 500nM or DMSO for an additional 17 days, and the emergence of a latently infected population (GFP-) was measured by flow cytometry over time. (**G**). CEM5.25 cells were pretreated with EPZ-719 at 500nM or control (DMSO) for three days, then infected with the replication competent HIV strain NL4-3. Infected cells were then cultured in the presence of EPZ-719 or control for an additional four days. At indicated timepoints, the fraction of infected cells (GFP+) was measured by flow cytometry. Each datapoint represents the average of triplicate samples. Error bars represent the standard deviation of the mean. Asterisk represents a comparison where P<0.05 (T Test).

To determine the impact of H3K36me3 depletion on HIV infection, we pretreated Jurkat cells with EPZ-719 for three days (experimental design shown in **Fig 1A**). Upon confirmation that these cells were depleted of H3K36me3 by western blot analysis (**Fig 1B**), we then infected the EPZ-719 exposed cells (or control vehicle exposed cells) with a GFP-expressing clone of HIV (HIV-dreGFP). This specific clone contains a short half-life GFP (dreGFP) within the envelope open reading frame, allowing dynamic analysis of LTR-driven HIV expression [37]. Productive HIV infection (%GFP+) was measured by flow cytometry at 3dpi, which showed similar levels of overall infection (29% vs 26%) for EPZ-719-treated cells and cells treated with control vehicle (DMSO), respectively (**Fig 1C and 1D**). These results suggest that SETD2/H3K36me3 is not required for HIV infection.

We next examined the impact of H3K36me3 on HIV latency. To do this, we exposed Jurkat cells to EPZ-719 for 3 days, infected with HIV-dreGFP, then flow sorted GFP+ cells at 3dpi to obtain an enriched actively infected population. We then cultured this infected population in the continuous presence of EPZ-719 or control vehicle for an additional 17 days. During this time, a minor subset of the actively infected cells lost viral gene expression and became GFP negative (GFP-), representing a latently infected population. Interestingly, we observed that EPZ-719 exposure significantly impacted the emergence of latently infected cells in the culture. For control treated cells, ~13% of infected cells cultured in the presence of control vehicle were GFP- by 8 days post infection (dpi). By contrast, EPZ-719 exposed cultures showed a substantially larger fraction of cells that lost GFP expression (~30%) (**Fig 1E and 1F**), indicating an increased frequency of latently infected cells in the culture. This difference in viral gene expression was maintained at least until 20dpi when the experiment was ended. EPZ-719 did not have any apparent impact on Jurkat cell viability or growth (**S2 Fig**). We also examined the impact of EPZ-719 on a spreading infection for a replication competent HIV strain (NL4-3). To study spreading infection, we used a clone of CEM T cells (5.25) that contains an integrated LTR-GFP cassette and which becomes GFP+ following productive infection due to *trans*-activation of the reporter cassette by viral expression of Tat [38]. CEM 5.25 cells were pretreated with EPZ-719 for 3 days then infected with NL4-3. After infection with the NL4-3 strain of HIV, we observed rapid viral spread throughout the cell culture indicated by rising GFP+ levels up to 3dpi, followed by a decline as infection saturates. For EPZ-719 exposed cells, spread of the virus was somewhat slower, with reduced levels of GFP+ cells at 2dpi and 3dpi, although ultimately, the virus was still able to spread and overtake the culture (**Fig 1G**). Together, these data are consistent with a model in which SETD2/H3K36me3 is not required for HIV infection but regulates post-integration HIV expression and latency.

### Inhibition of HIV expression by EPZ-719 is reversible

**Fig 2:**
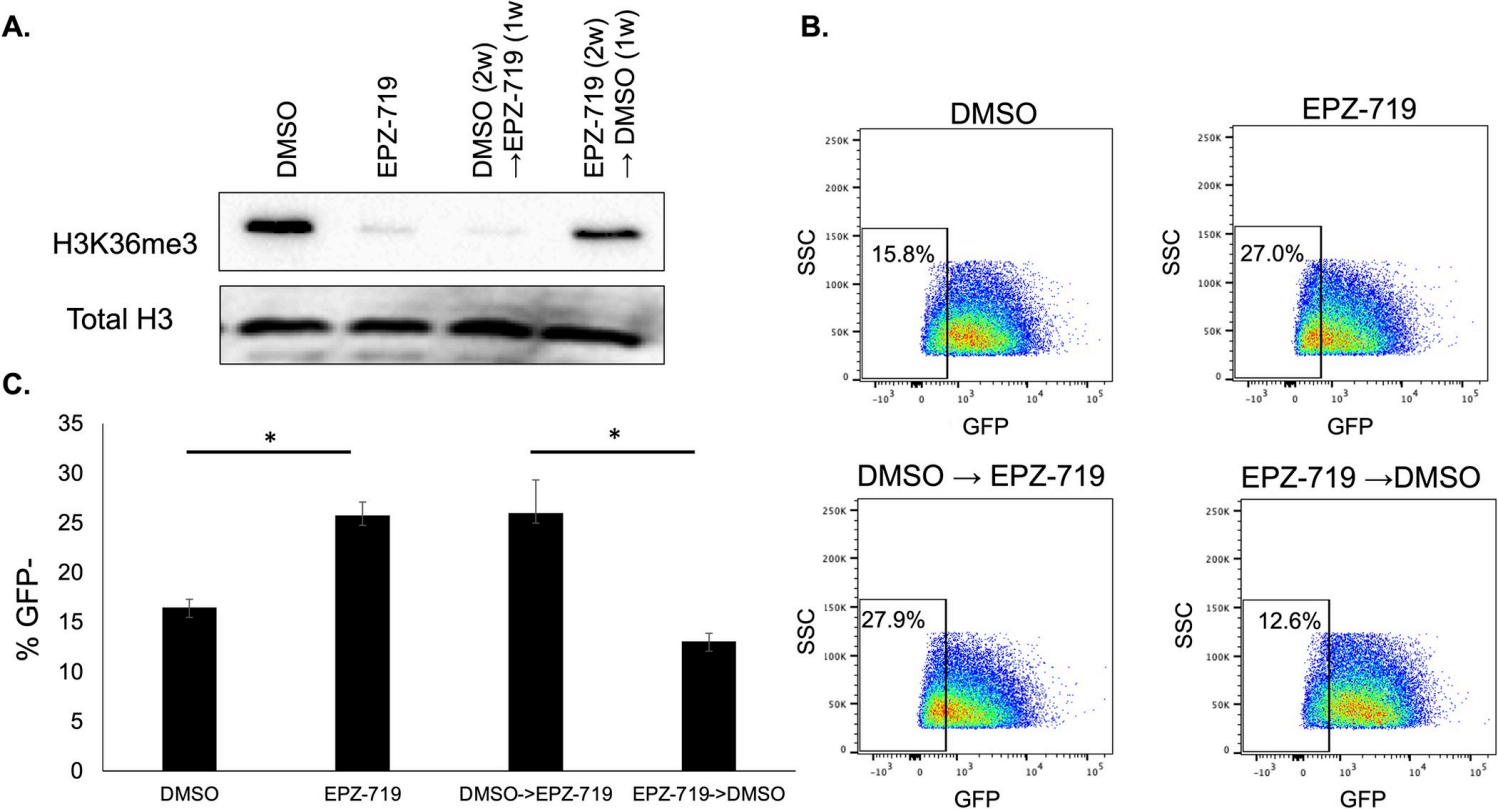
EPZ-719 inhibition of HIV expression is reversible. Jurkat cells were pretreated with EPZ-719 at 500nM or DMSO for three days, then infected with HIV-dreGFP. At 2dpi, productively infected cells (GFP+) were enriched by flow sorting, then cultured for an additional two weeks in EPZ-719 or DMSO. At 2wpi the culture was divided and exposure conditions were reversed for a subset of the cells before an additional week of culture. At 3wpi, the level of H3K36me3 and total H3 was measured by western blot (**A**), and viral gene expression measured by flow cytometry (**B, C**). Each datapoint represents the average of triplicate samples. Error bars represent the standard deviation of the mean. Asterisk represents a comparison where P<0.05 (T test).

Since H3K36me3 associates with LEDGF, an HIV integrase interacting factor, we hypothesized that the increased post-integration silencing of HIV in the absence of H3K36me3 might result from a difference in integration sites. If this were true, then the impact of EPZ-719 on HIV expression from treatment prior to infection should persist even if the drug is removed after viral integration. Similarly, we would predict that HIV expression in infected cells would be resistant to EPZ-719 post integration. We therefore examined whether depletion of H3K36me3 in HIV infected cells induces an ongoing state of transcriptional repression even after withdrawal of the inhibitor. To test this hypothesis, we pretreated Jurkat cells with DMSO control or EPZ-719 for three days to deplete H3K36me3, then infected with HIV-dreGFP, before sorting infected (GFP+) cells. The infected cells were then maintained in EPZ-719 or DMSO for two weeks, to allow a latently infected (GFP-) population to emerge. As expected, EPZ-719 exposed cells exhibited a higher proportion of latently infected (GFP-) cells and reduced levels of H3K36me3 (**Fig 2A–2C**). At 2 weeks post infection (wpi), we then divided the culture, and added EPZ-719 to cells that had previously only been exposed to DMSO and removed EPZ-719 from the cells that had been exposed to that, replacing with DMSO. The cells were then maintained in their new condition for 1 week, before re-measuring viral gene expression by flow cytometry (**Fig 2B and 2C**). Notably, when we examined H3K36me3 levels after this 1w exposure, the cells had altered their H3K36me3 levels to match their new condition–newly EPZ-719-exposed cells having lost H3K36me3, and cells that had EPZ-719 removed having restored H3K36me3 levels. In both cases, the infected cells had also altered viral gene expression to match the new exposure–cells that were switched from DMSO to EPZ-719 showed reduced viral expression and elevated latency, while cells that were switched from EPZ-719 to DMSO restored viral gene expression to normal levels. Thus, we conclude that inhibition of HIV expression by EPZ-719 is reversible, along with H3K36me3 levels within the infected cells. This conclusion is inconsistent with inhibition of integration or altered integration site locations contributing to the observed viral suppression in EPZ-719 treated cells. These observations also rule out the possibility that loss of GFP expression in EPZ-719 is due to progressive loss of unintegrated viral DNA over time since this phenomenon would not be reversible.

### H3K36me3-modified histones are present across the HIV genome and are depleted by EPZ-719 treatment

**Fig 3:**
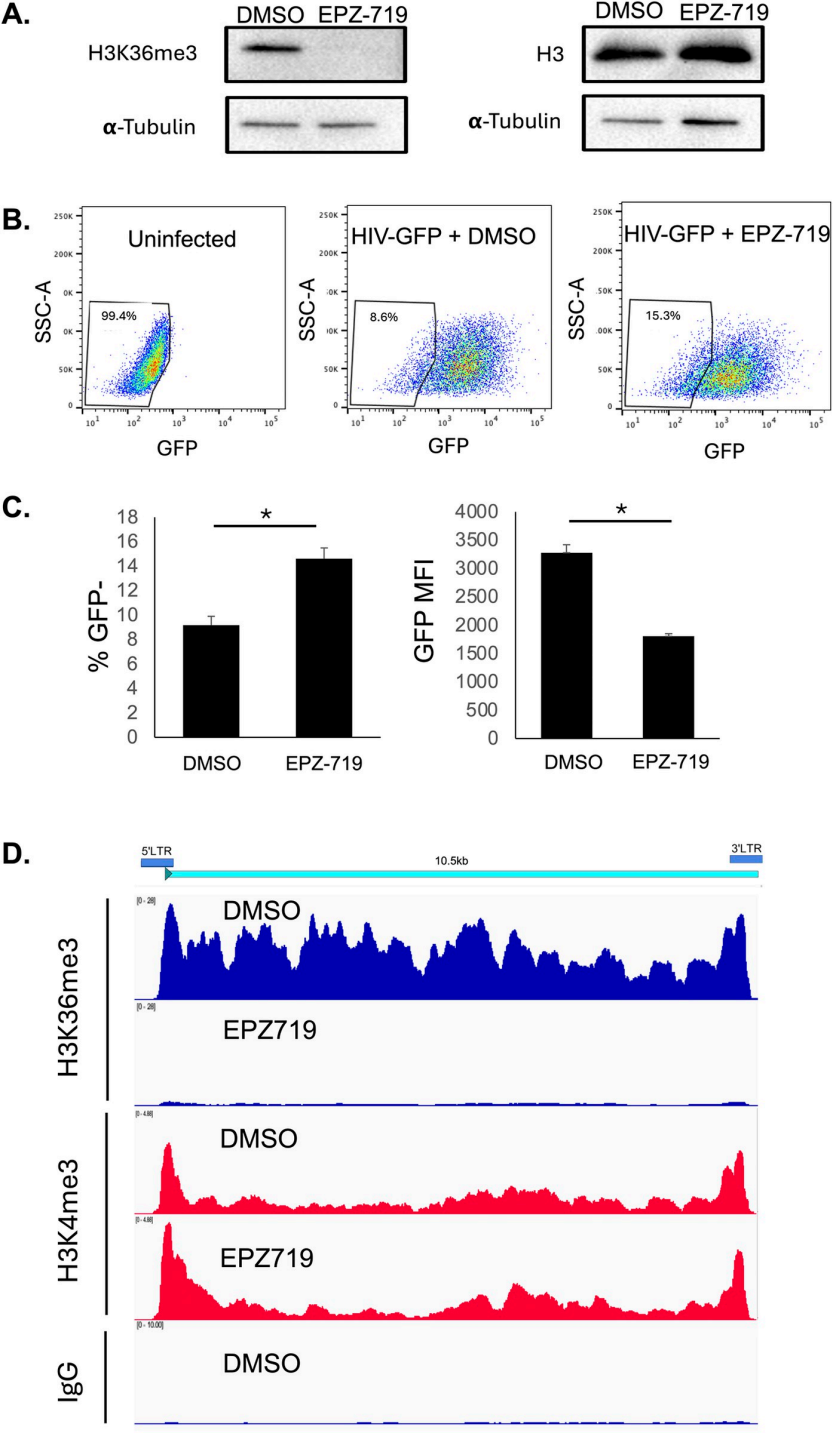
H3K36me3-modified histones are present across the HIV proviral genome and are depleted by EPZ-719 exposure. Jurkat cells were infected with HIV-dreGFP then sorted to enrich for actively infected (GFP+) cells. Infected cells were then cultured in the presence of EPZ-719 at 500nM or control vehicle (DMSO) for 8 days, then cellular protein abundance was analyzed by western blot (**A**) and viral gene expression measured by flow cytometry (**B**). The cells were then analyzed by Cleavage Under Targets & Release Using Nuclease (CUT&RUN) using antibodies against H3K36me3, H3K4me3 and control IgG. (**C**) Normalized coverage of sequencing reads across the HIV reference genome is displayed using Integrative Genomics Viewer version 2.17.

Although our data indicate that EPZ-719 causes a global depletion of H3K36me3 in exposed cells, we next examined whether H3K36me3 could be observed at the HIV provirus, and whether EPZ-719 affects the level of HIV-associated H3K36me3. We first infected Jurkat cells with HIV-dreGFP, then enriched actively infected (GFP+) cells by flow sorting and exposed the infected cells to EPZ-719 for 8 days. As expected, EPZ-719 caused a strong reduction in the global level of H3K36me3 in the infected cells (**Fig 3A**) and reduced the level of HIV expression compared to vehicle exposed control cells (**Fig 3B and 3C**). To examine histone modifications at the HIV provirus, we then used Cleavage Under Targets and Release using Nuclease (CUT&RUN) [39]. This assay involves staining chromatin with an antibody against a primary target followed by a secondary stain with Protein A that is fused to micrococcal nuclease (MNase), releasing fragments that are proximal to the antibody target. Next generation sequencing of these fragments can then be used to map protein/DNA interactions across the genome. From alignment of the CUT&RUN sequencing data to the HIV genome, we observed that, in infected cells exposed to empty vehicle (DMSO), H3K36me3 was abundantly present across the entire proviral genome (**Fig 3D**). In contrast, H3K4me3, which typically marks active promoters, was found mostly at the viral LTR. EPZ-719 exposure caused a strong reduction in H3K36me3 occupancy across HIV, consistent with its global effects, while H3K4me3 was not reduced. Overall, these data confirm that HIV proviruses are directly associated with SETD2-mediated H3K36me3 marks across the entire viral genome, and that EPZ-719 depletes H3K36me3 at the HIV provirus in addition to its global effects.

### H3K36me3 depletion inhibits HIV expression in primary CD4 T cells

**Fig 4:**
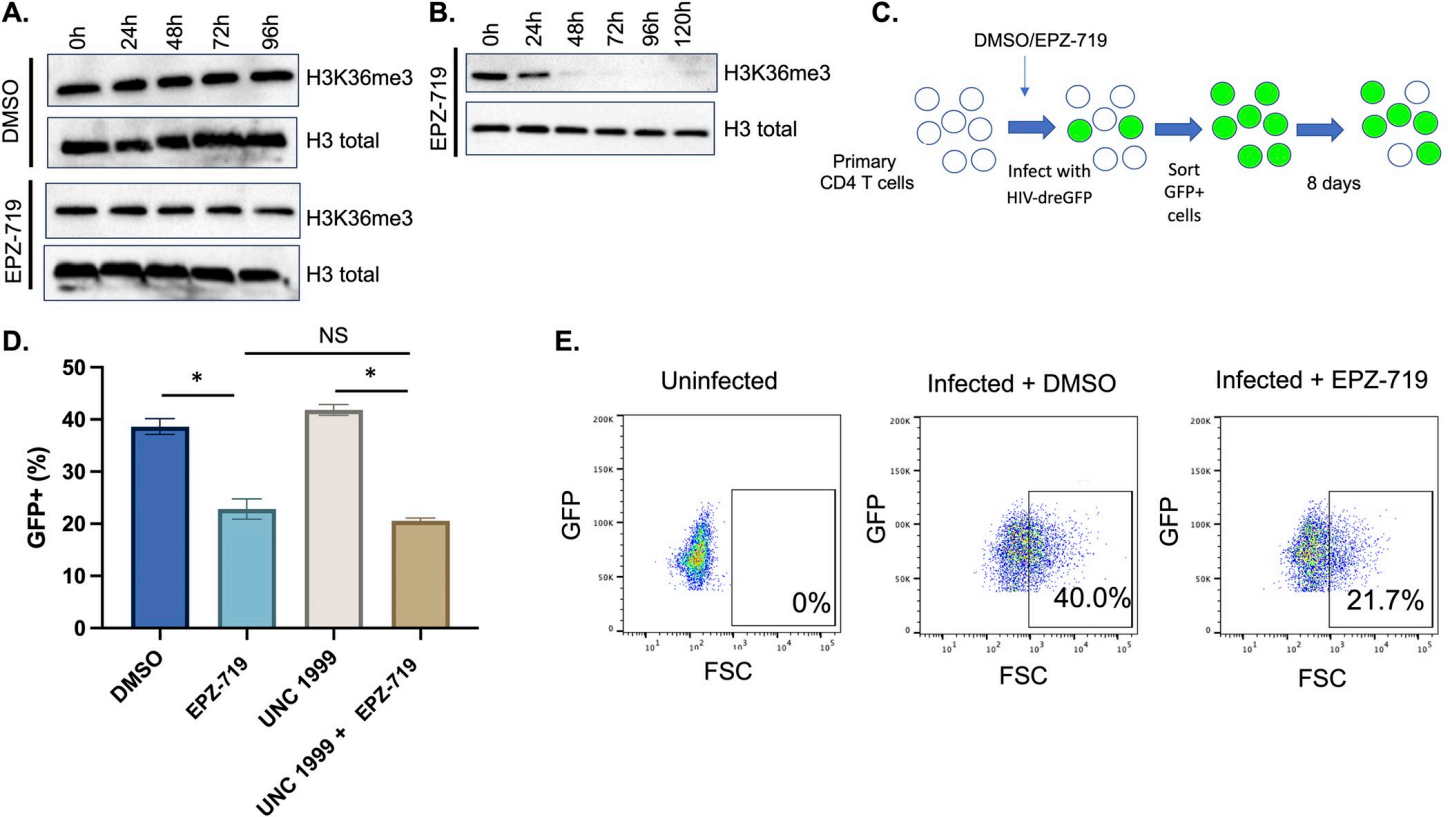
SETD2 activity is required for HIV expression in primary CD4 T cells. 500nM EPZ-719 was added to resting (**A**) or activated (**B**) CD4 T cells for the length of time indicated. Total protein extracts were then harvested and western blotted for H3K36me3 and total histone H3. (**C**) Schematic overview of primary cell model of HIV latency. CD4 T cells were activated for 2d, then exposed to EPZ-719 or DMSO for an additional 2d before being infected with HIV-dreGFP. At 2dpi, GFP+ cells were then flow sorted and cultured for an additional 8 days in the presence of EPZ-719 (500nM), UNC1999 (1μM), EPZ-719+UNC1999 or DMSO. Viral gene expression was then measured by flow cytometry and the percentage of cells with active expression (GFP+) calculated (**D, E**). Each datapoint represents the average of triplicate samples. Error bars represent the standard deviation of the mean. Asterisk represents a comparison where P<0.05 (T Test).

Although Jurkat cells are a valuable model for HIV infection, the natural target cell for HIV is primary CD4 T cells, and several differences exist between primary CD4 T cells and clonal immortalized T cell lines. Thus, we investigated whether our observations regarding the impact of EPZ-719 and H3K36me3 depletion in Jurkat cells would extend to primary CD4 T cells. We first examined whether exposure of primary CD4 T cells to EPZ-719 led to depletion of H3K36me3. Notably, we observed no significant reduction of H3K36me3 levels in resting CD4 T cells, even after 4 days of exposure (96h) to EPZ-719 (**Fig 4A**). This suggests that, in resting CD4 T cells, SETD2 activity is not required to maintain H3K36me3 over this period of time, likely due to a low rate of removal of this mark and/or low levels of cell division needed to dilute it. By contrast, when we added EPZ-719 to recently activated primary CD4 T cells, we observed rapid loss of H3K36me3, with a clear reduction apparent by 24h and near complete loss of H3K36me3 by 48h of exposure (**Fig 4B**). This difference in potency of EPZ-719 between resting and primary CD4 T cells is likely due to more rapid removal of H3K36me3 in activated cells and higher levels of cell division, thereby requiring SETD2 activity to maintain H3K36me3 levels. Thus, we focused on the impact of EPZ-719 on HIV infection in activated primary CD4 T cells. To investigate the role of SETD2/H3K36me3 in activated primary CD4 T cells, we used a primary CD4 T cell model of HIV infection and latency that we have previously established (**Fig 4C**) [16,37,40]. In this model, primary CD4 T cells are activated for two days then infected with HIV-dreGFP. Actively infected cells (GFP+) are then enriched at 2dpi by flow sorting and cultured for an extended period of time. Infected cells progressively reduce HIV expression as they return to a resting state and latently infected cells (GFP-) emerge over the course of several days. When we combined this model with exposure to EPZ-719 or control vehicle (DMSO) we observed a significant reduction in the percent GFP+ cells for the EPZ-719 exposed cells compared to control cells (22% GFP+ vs 40% GFP+) at 8dpi (**Fig 4D and 4E**). This change was not associated with an impact of EPZ-719 on expression of activation markers (CD38, HLA-DR) (**S3 Fig**). These data indicate that the impact of EPZ-719 and the role of SETD2 in HIV expression is conserved in primary CD4 T cells. Some prior data has indicated that SETD2 can help to promote gene expression by blocking silencing by the Polycomb-repressive complex 2 (PRC2) that generates the H3K27me3 histone mark [41]. To investigate this possibility, we examined an inhibitor of EZH2, the catalytic subunit of PRC2 (UNC1999) in parallel, and in combination with, EPZ-719 and found no impact of UNC1999 on HIV expression or on inhibition of HIV by EPZ-719. Thus, inhibition of HIV expression by EPZ-719 was not dependent on EZH2/PRC2 activity.

### SETD2 expression is required for HIV expression in primary CD4 T cells

**Fig 5:**
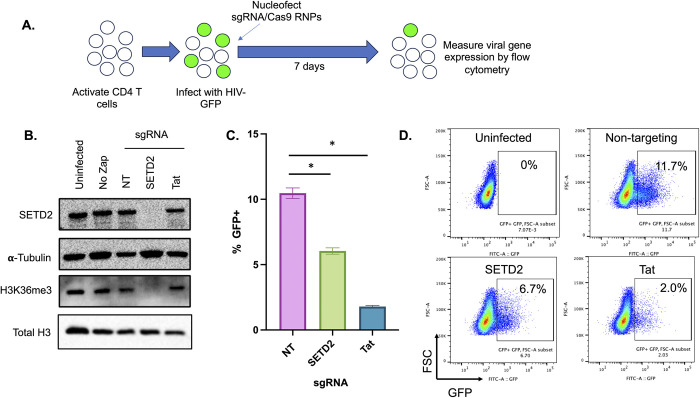
SETD2 knockout in primary CD4 T cells reduces HIV expression. (**A**). Schematic overview of SETD2 knockout in primary CD4 T cells. CD4 T cells were activated for 2 days, then infected with HIV-dreGFP. At 2dpi, cells were nucleofected with ribonucleoparticles (RNPs) targeting SETD2, Tat, or non-targeting (NT) control. At 8d post targeting, total cell protein lysate was extracted and western blotted for SETD2, H3K36me3, total histone H3 and α-tubulin (**B**). Uninfected cells and infected but not nucleofected cells (“No Zap”) are also shown. Viral gene expression was measured by flow cytometry (**C,D**). Each datapoint represents the average of triplicate samples. Error bars represent the standard deviation of the mean. Asterisk represents a comparison where P<0.05 (T test).

To further confirm the role of SETD2 in HIV expression in primary CD4 cells, we performed a CRISPR/Cas9-mediated knockout of SETD2 in HIV infected cells. To achieve this, ribonucleoprotein (RNP) complexes consisting of Cas9 and crRNAs targeting SETD2, the viral transcriptional regulator Tat, or a non-targeting (NT) control were nucleofected into activated HIV-dreGFP infected primary CD4 T cells at 2dpi (**Fig 5A**). Western blot confirmed loss of SETD2 expression in the cells and reduced H3K36me3 levels (**Fig 5B**). When we examined viral gene expression at 8dpi, we observed a significantly reduced level of GFP+ cells in the SETD2 targeted cells compared to cells nucleofected with non-targeting control RNPs. Importantly, targeting of Tat led to a strong reduction in viral gene expression, confirming that this approach can detect regulators of HIV expression (**Fig 5C and 5D**). Overall, these data provide further confirmation that SETD2 is an important regulator of HIV expression in primary CD4 T cells and that SETD2 activity is required to maintain active HIV gene expression in CD4 T cells.

### H3K36me3 loss affects cellular gene expression but does not alter HIV RNA levels

**Fig 6:**
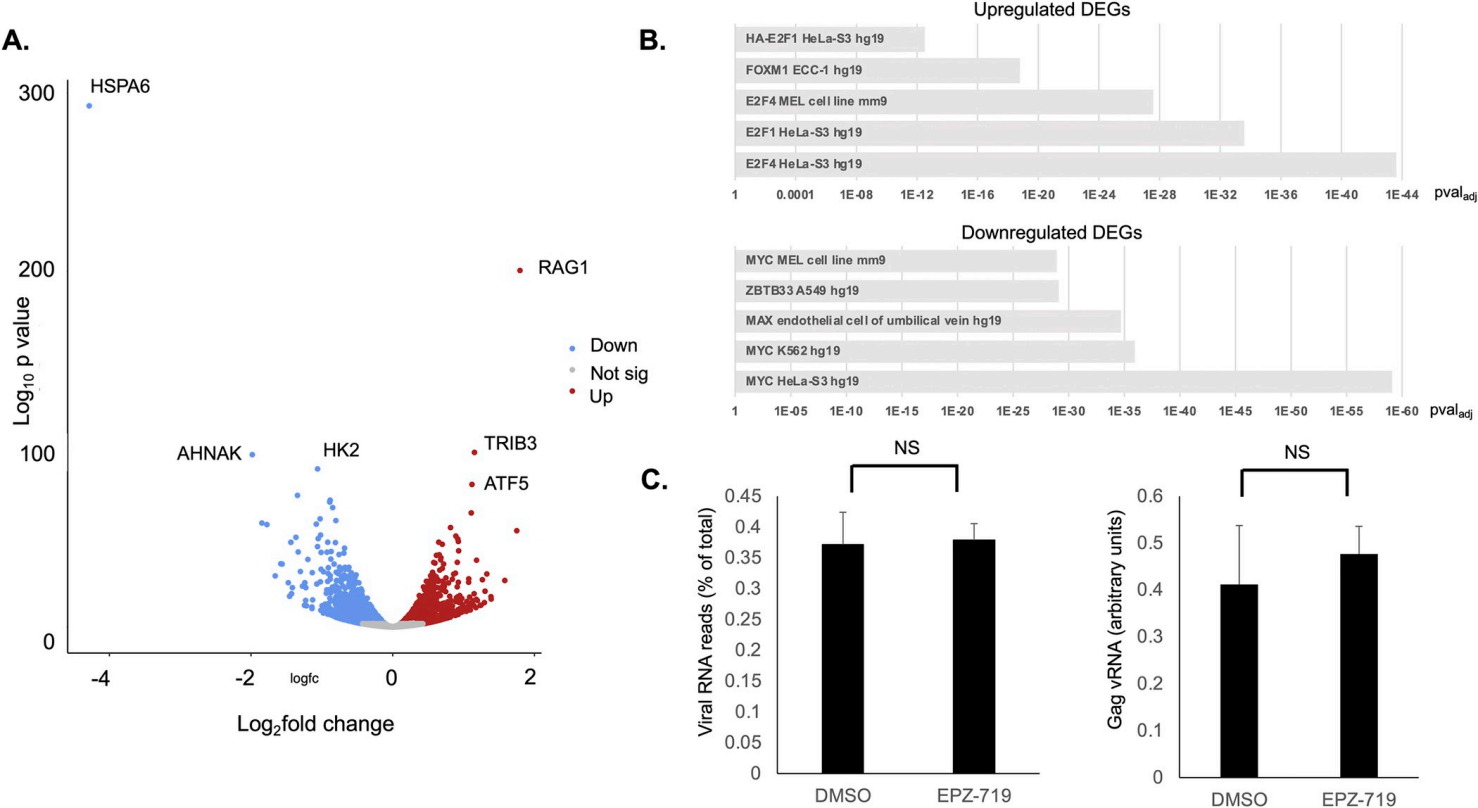
EPZ-719 affects cellular gene expression but not HIV RNA levels. HIV-dreGFP infected Jurkat cells that had been exposed to EPZ-719 (500nM) or DMSO for three days before infection and 8 days after infection, were profiled by bulk RNAseq. (**A).** Volcano plot of differentially expressed genes (DEGs), with significantly downregulated (pval_adj_<0.05) genes in blue and upregulated genes shown in red. The three most significantly upregulated and downregulated genes are labeled. Not sig = not significantly different (grey). (**B).** The upregulated and downregulated genes were analyzed using Enrichr to find enrichment of known protein/DNA binding sites from the ENCODE database within the gene set. (**C).** The abundance of unique viral reads as a percentage of total unique reads in the dataset was calculated (left panel), and the abundance of Gag viral RNA (Gag-vRNA) was also examined by quantitative PCR (right panel, arbitrary units). The analysis was performed on biological triplicate samples. Error bars represent the standard deviation of mean. NS = not significant, T test (P>0.05).

To further understand the role of SETD2 in regulation of HIV expression, we performed RNAseq on HIV-dreGFP infected Jurkat cells that had been cultured in the presence of EPZ-719 or control vehicle (DMSO). Interestingly, we observed many differentially expressed genes (DEGs) between the two conditions, 4833 genes total (pval_adj_<0.05), with 2310 genes upregulated and 2523 downregulated (**Fig 6A, S1 Table**). However, most of these changes were modest in magnitude with only 34 upregulated DEGs and 38 downregulated DEGs changing by greater than log_2_FC>1. Nevertheless, we observed some intriguing patterns in the transcriptomic changes. When we examined upregulated genes for enrichment with particular chromatin features using Enrichr [42], we observed a strong enrichment for genes that are associated with E2F binding (**Fig 6B, upper panel, S2 Table**). By contrast, downregulated genes were highly associated with Myc binding peaks (**Fig 6B lower panel, S3 Table**). Notably Myc expression itself was reduced by EPZ-719 exposure, consistent with an overall reduction in Myc-dependent gene expression. We also performed gene ontology (GO) analysis [43] of the top 500 upregulated or downregulated genes and observed that upregulated DEGs were highly enriched for structural constituents of chromatin (GO:0030527) (p_adj_ = 7.96x10^-14^) while downregulated genes were highly enriched in RNApol2 specific DNA-binding transcription factor activity (GO:0003700, p_adj_ = 3.55x10^-20^) (**S4–S7 Tables**). These observations are consistent with EPZ-719 having a broad effect on cellular chromatin and RNApol2 driven transcription.

We also examined expression of several known regulators of HIV transcription in the RNAseq dataset. We previously identified the chromatin insulator CTCF as inhibiting HIV expression in infected cells [16], and CTCF expression was upregulated by EPZ-719 exposure. Expression of the catalytic subunit of the Polycomb Repressive Complex 2 (PRC2) EZH2 was also increased in EPZ-719 exposed cells. Interestingly, we observed some evidence of innate immune activation in EPZ-719 exposed cells–expression of the interferon regulated genes IRF1, IRF4, MX2, IFIT3, and IFIT2 were all upregulated. Additionally, cytokine signaling mediators STAT4 and STAT2 were both upregulated in response to EPZ-719. We also examined the expression of HIV restriction factors in the dataset and observed mixed outcomes. APOBEC3F, APOBEC3D, as well as APOBEC2 were all upregulated in the presence of EPZ-719. By contrast, BST2, SERINC1, SERINC2, and SERINC5 were downregulated. Overall, these data indicate that H3K36me3 loss affects a broad number of cellular genes, including several that could have a direct or indirect impact on HIV expression.

To investigate whether H3K36me3 loss affects the HIV RNA expression level, we also compared the overall fraction of HIV mapping reads in the EPZ-719 exposed cells to the control cells. Interestingly, the abundance of HIV reads was similar between the two conditions indicating no impact of EPZ-719 on overall HIV RNA levels (**Fig 6C, left panel**). Additionally, visualization of HIV mapping reads across the viral genome showed a similar overall level of distribution for both conditions (**S4 Fig**). To confirm this observation, we carried out quantitative real-time PCR analysis of Gag RNA levels in HIV-dreGFP infected Jurkat cells that had been cultured in the presence of EPZ-719 or DMSO for 8 days (**Fig 6C right panel**). Similar to the RNAseq results, we found no significant difference in the levels of Gag RNA with EPZ-719 exposure, although we did observe a trend towards increased Gag RNA in EPZ-719 exposed cells relative to control exposed cells. This observation suggests that EPZ-719 does not reduce the overall level of HIV transcription and RNA abundance, but instead may be impacting a post-transcriptional step in HIV expression. We further examined this hypothesis by quantifying the abundance of nascent viral RNAs in infected cells using 5-ethnyluridine labeling followed by biotinylation and isolation of labeled RNAs from cells exposed to DMSO or EPZ-719 for 8 days. Consistent with our previous results, we observed no change to viral RNA levels in total RNA in the presence of EPZ-719 (**S5 Fig, panel A**). When we examined nascent HIV transcripts, we observed no clear change to abundance (**S5 Fig, panel B**). We also examined the impact of EPZ-719 on Gag RNA levels in HIV-dreGFP infected primary CD4 T cells that had been exposed to EPZ-719 or DMSO for 7 days and observed no difference between the conditions (**S5 Fig, panel C**). Together these data suggest a model in which EPZ-719 inhibits HIV expression at a post-transcriptional step. Nevertheless, it remains possible that EPZ-719 impacts an aspect of HIV transcription not measured by these assays.

### SETD2/H3K36me3 affects HIV RNA splicing

We next considered post-transcriptional mechanisms by which EPZ-719 might affect HIV expression. H3K36me3 has been shown to regulate m6A modification of RNA by METTL3 [44], and m6A RNA methylation significantly impacts HIV expression and replication [45–48]. We therefore examined total cellular m6A levels in cellular RNA after 13d of exposure of HIV-dreGFP infected cells to 500nM EPZ-719 **(S6 Fig, panel A**). After this period of culture, EPZ-719 exposed cells exhibited reduced HIV expression as expected. However, despite a clear difference in the expression of HIV and the abundance of latently infected (GFP-) cells in the culture (**S6 Fig, panel B**), we observed no change in total m6A abundance compared to DMSO control exposed cells (**S6 Fig, panel C**). To examine m6A RNA modification within HIV transcripts, we also performed immunoprecipitation of m6A RNA from infected cells, followed by quantitative real time PCR for a region of HIV within the Env/Rev coding sequence that has been shown to be abundantly modified with m6A [46,47]. As expected, m6A-specific pull down significantly enriched this RNA sequence over control IgG, but we observed no difference in abundance between EPZ-719 exposed cells and control (DMSO) exposed cells (**S6 Fig, panel D**). Thus, we conclude that EPZ-719 likely does not impact HIV expression through affecting m6A modification of viral or cellular RNAs.

**Fig 7:**
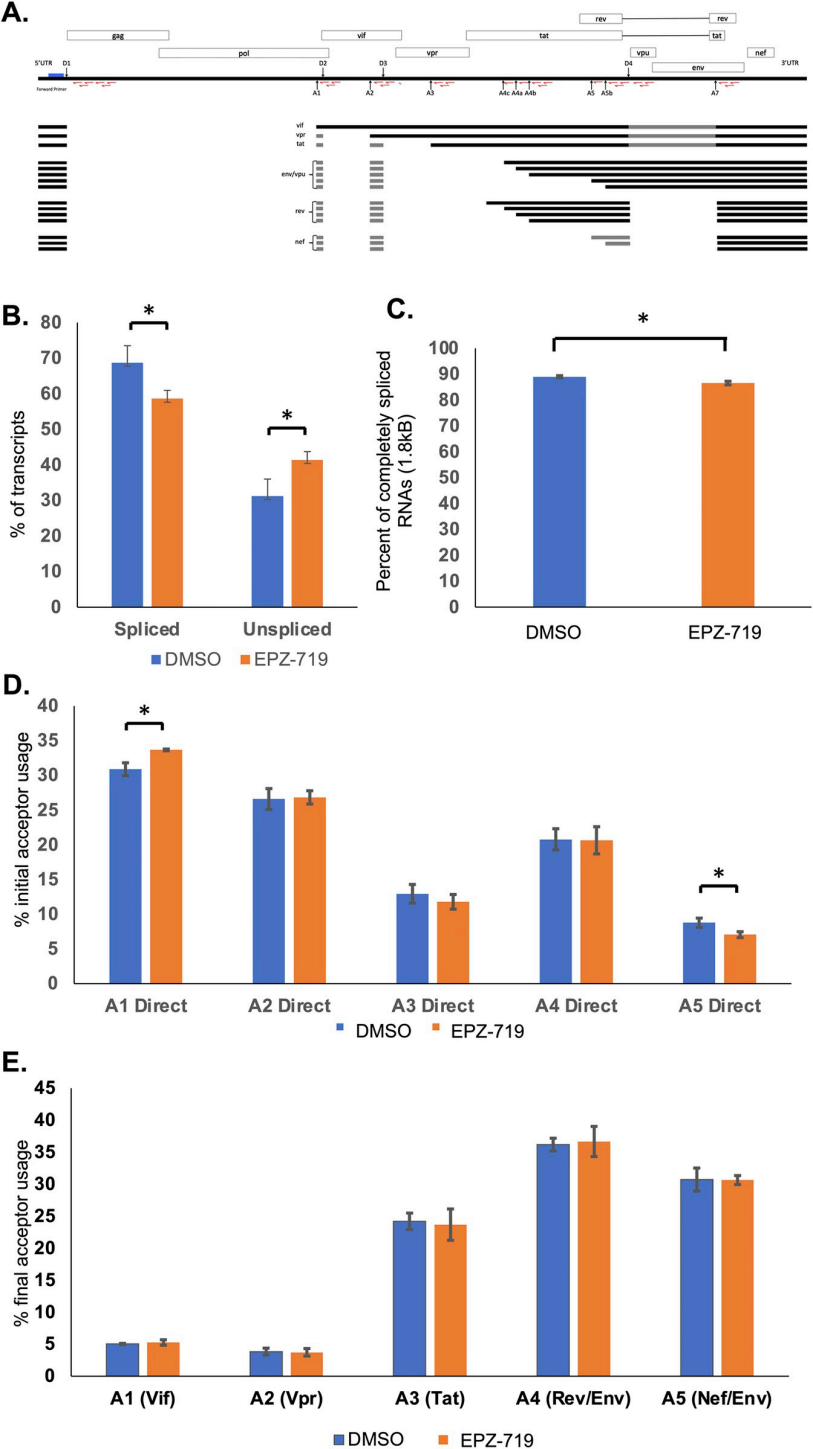
EPZ-719 affects HIV RNA splicing. HIV RNA splicing was quantified by a method in which primers including a random barcode sequence of nucleotides were used for reverse transcription of RNA from HIV-dreGFP infected Jurkat cells exposed to EPZ-719 or DMSO, followed by PCR for the major 4kb and 1.8kb species of viral RNA, and Illumina sequencing. (**A**). The location of the primer binding sites and the HIV slice donor (D1-D4) and acceptor (A1-A7) sites are shown on a map of the HIV genome. (**B**). The fraction of vRNAs that are spliced is shown. (**C**). The percentage of completely spliced vRNAs (1.8kb) is shown. (**D**). The fraction of spliced transcripts using different initial acceptors is shown. (**E**). The fraction of spliced transcripts using different final acceptors is shown. Each bar represents the average of three independent biological replicates. Error bars represent the standard deviation of the mean. Asterisk indicates significant difference (P<0.05, Students T test).

We next considered whether SETD2 might be impacting HIV transcript splicing. The full-length HIV genomic transcript can be extensively spliced to generate up to 50 different RNA forms, using four splice donor sites (D1, D2, D3, D4) and ten different acceptor sites (A1, A2, A3, A4a, A4b, A4c, A4d, A5, A5b, A7) (**Fig 7A**). To investigate this, we used a recently developed assay to quantify individual HIV RNA species in HIV-dreGFP infected cells after 13d of exposure to EPZ-719 at 500nM [49]. This assay involves first performing a reverse transcription reaction with a random unique molecular identifier that also serves as a primer to capture all HIV RNA species—unspliced, 4kb, and 1.8kb. During reverse transcription the random unique molecular identifier is added to the cDNA, allowing accurate quantification of each molecule in the original pool after subsequent PCR amplification and Illumina sequencing. After performing this analysis, we observed that in the EPZ-719 treated cells, there was a small but significant overall reduction in the fraction of spliced transcripts compared to the control cells (59% vs 69%) (**Fig 7B**). Additionally, for the EPZ-719 treated cells, there was a small reduction in the percentage of 1.8kb transcripts as a fraction of the spliced transcripts (**Fig 7C**). Furthermore, when we examined splice donor and acceptor usage within the spliced transcripts, we noticed a small increase in usage of the A1 initial acceptor and a reduction in the A5 initial acceptor usage (**Fig 7D**). However, when we compared the final acceptor usage, there was no significant difference between the two conditions (**Fig 7E**). To confirm these results in primary CD4 T cells, we infected activated primary CD4 T cells with HIV-dreGFP then cultured in the presence of EPZ-719 at 500nM for eight days, before analysis of HIV transcript splicing using the same sequencing-based assay we had used for Jurkat cells. Similar to what we observed for Jurkat cells, we observed a significant decrease in the overall percentage of HIV RNAs that were spliced in the presence of EPZ-719 (**S7 Fig, panel A**), while there was no change to the percentage of spliced viral RNAs that were fully spliced, and no apparent change to the initial or final splice acceptor sites used (**S7 Fig, panels B-D**). We also examined the impact of EPZ-719 on the splicing of cellular transcripts using the RNAseq dataset we had generated from EPZ-719 exposed HIV infected Jurkat cells. We observed 1399 cellular genes with differential exon usage in the presence of EPZ-719 (**S8 Fig**, **S8 Table**). Overall, these observations indicate that loss of H3K36me3 can alter viral and cellular splicing in infected cells and leads to a modest but significant reduction in spliced viral RNAs. This effect on viral splicing may contribute to the overall suppressive effect of EPZ-719 on viral expression.

### Impact of EPZ-719 on HIV integration sites

**Fig 8:**
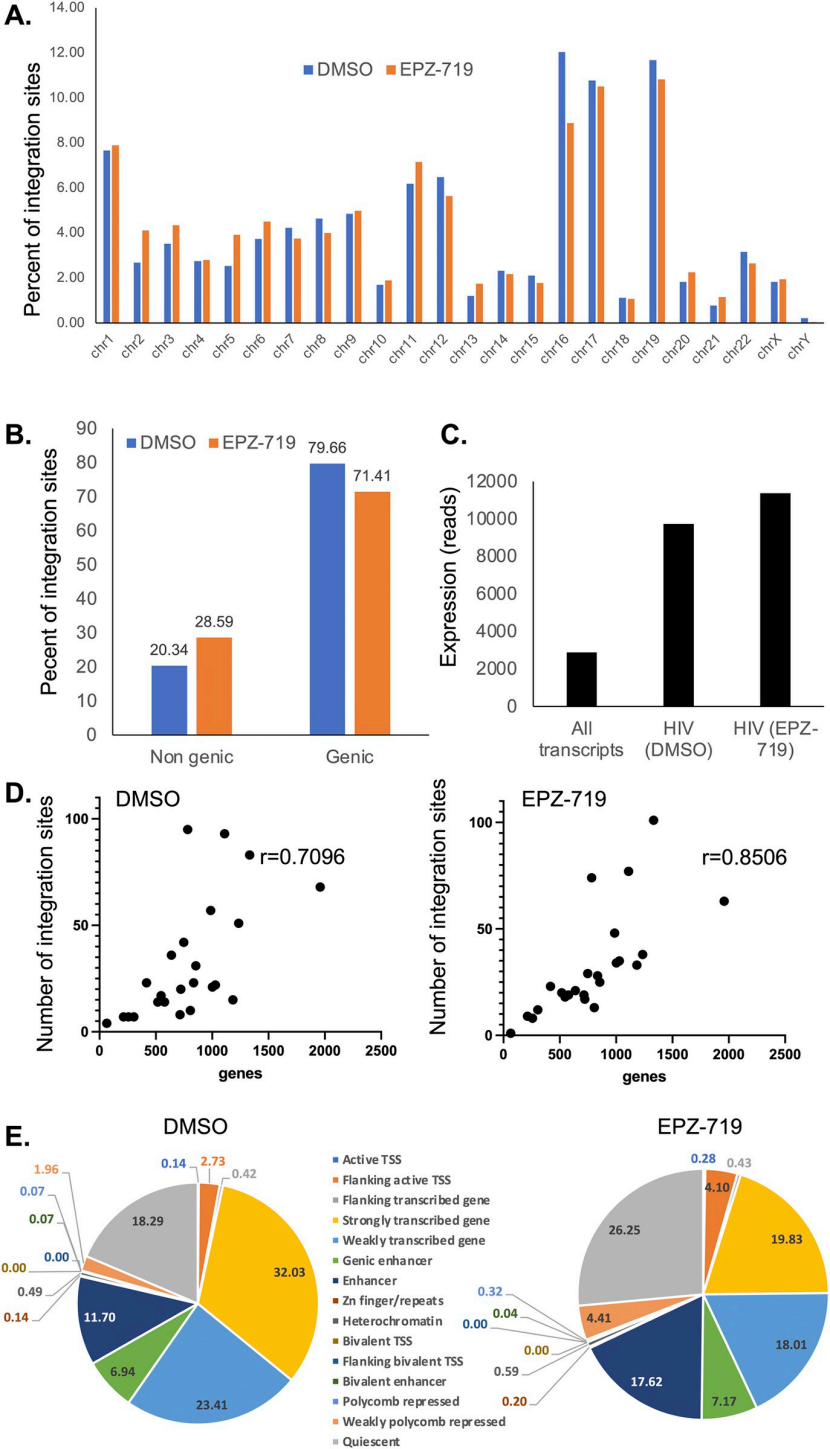
Analysis of HIV integration sites in the presence of EPZ-719. Genomic DNA derived from Jurkat cells infected with HIV-dreGFP in the presence of EPZ-719 (500nM) or control (DMSO) was used to identify 2531 and 1421 integration sites respectively. For these integration sites, we compared their chromosomal distribution (**A**), their association with genes (**B**), the normalized expression level of cellular genes associated with HIV integration sites that were infected in the presence of EPZ-719 or DMSO control (**C**), and the Spearman correlation between the number of genes and the number of integration sites across chromosomes (**D**). (**E**). Each integration site was annotated as belonging to one of 15 different chromatin/transcriptional environments using ChromHMM.

To further examine whether SETD2 activity or H3K36me3 levels in target cells impacted HIV integration, we extracted cellular genomic DNA from Jurkat cells that had been exposed to EPZ-719 or control (DMSO) for 3 days then infected with HIV-dreGFP. Integration sites from each condition were then determined by linear amplification from the HIV LTR followed by nested PCR and long read sequencing of the amplified product [50]. Using this approach, we recovered HIV integration sites from cells in either the DMSO or EPZ-719 condition (1421 and 2531 respectively). Examination of the integration site distribution across the genome showed, consistent with previous reports [51], that the distribution of integration sites across the chromosomes was non-random, with certain chromosomes such as Ch19 having elevated enriched abundance of integration sites (**Fig 8A)**. When we compared integration sites between cells infected in the presence of EPZ-719 to cells infected in the presence of DMSO, we observed an overall similar distribution across the chromosomes, with only minor differences apparent (**Fig 8A**). Some chromosomes such as Ch16 exhibited apparent differences in the proportion of integrants for EPZ-719-exposed cells (12.0% for DMSO versus 8.9% for EPZ-719). The significance of this difference is unclear. We then examined the association of the integration sites with genes. As expected, the majority of HIV integration sites (80%) were associated with genes rather than intergenic regions (20%) for cells infected in the presence of DMSO (**Fig 8B**). This observation was also true for EPZ-719 exposed cells, with 71% of integration sites associated with genes and 29% within intergenic regions, although the data indicate a modest shift from integration in genic regions to non-genic regions in the presence of EPZ-719. We conclude from this that the association of HIV integration with genes does not require SETD2 activity or H3K36 methylation, but that SETD2 activity does influence the distribution of integration sites.

Since H3K36me3 is deposited by SETD2 during transcription and is associated with actively expressed genes, we also examined the expression level for genes that were associated with HIV integration sites in the presence of EPZ-719 or DMSO control using our RNAseq dataset. For both conditions, HIV integration was enriched in genes that were highly expressed in infected cells–the average normalized transcript read count for all genes was 2890, while, for genes associated with HIV integration sites in EPZ-719 or DMSO treated cells, the average expression level was 11337 and 9739 respectively (**Fig 8C**). Thus, EPZ-719 exposure and H3K36me3 depletion did not prevent the association of HIV integration with highly expressed genes. Additionally, we examined the correlation between the number of integration sites on each chromosome and the number of genes on that chromosome. We observed a strong positive correlation between the number of genes on a chromosome and the number of integration sites. This observation was true for both control cells and EPZ-719 exposed cells (r = 0.7096 and r = 0.8506 respectively) (**Fig 8D**). We also examined whether integration site (IS) genes were enriched in differentially expressed genes (DEGs) from our RNAseq dataset. We observed that the set of IS genes for both DMSO and EPZ-719 conditions contained a higher proportion of DEGs (35%) than the overall fraction of cellular genes that were DEGs (17%) (**S9 Fig, panel A**), indicating that integration sites are enriched in DEGs. When we compared the fold change and directionality of changes in gene expression for IS genes that were also DEGs, we observed that IS genes in DMSO and EPZ-719 exposed cells both exhibited a similar distribution of differential gene expression to the overall set of differentially expressed genes (**S9 Fig, panel B**).

To examine the impact of EPZ-719 on the HIV integration sites in more detail, we next used ChromHMM to annotate the chromatin “neighborhood” of each integration site [52]. This approach uses a hidden Markov model to categorize specific genomic locations based on the known abundance of sets of chromatin marks. Specifically, we defined each integration site as belonging to one of 15 different chromatin/transcription states. As expected, a large fraction of HIV integration sites were observed in chromatin domains associated with actively transcribed genes, with regions defined as have characteristics of weak transcription or strong transcription comprising 55% of the sites for control (DMSO) treated cells. Enhancer regions were also highly represented (**Fig 8E,** left panel). Notably, strongly transcribing gene regions exhibit high levels of H3K36me3 modification [52]. Additionally, 18% of integrations in the DMSO condition occurred in regions categorized as “quiescent”, with few histone modifications. In EPZ-719 exposed cells, these trends were also present, but we observed a shift in the representation of certain categories (**Fig 8E**, right panel). Interestingly, we observed that EPZ-719 caused a reduction in the association of HIV integration with highly transcribed gene regions (18.3% vs 30.8%), while integration into weakly transcribed gene regions was unaffected. Correspondingly, in EPZ-719 exposed cells we observed an increase in integration sites in genomic regions that were categorized as quiescent (26.3% vs 18.3%), as enhancers (17.6% vs 11.7%), or as weakly polycomb-repressed regions (4.4% vs 2.0%). Overall, these results indicate that in the absence of H3K36me3, HIV is still able to integrate in to host cell genome, and maintains a preference for actively transcribed genes, but exhibits a redistribution away from highly transcribed chromatin regions in favor of quiescent regions, enhancers and polycomb-repressed regions.

### LEDGF associates with cellular chromatin in a H3K36me3-independent manner

**Fig 9:**
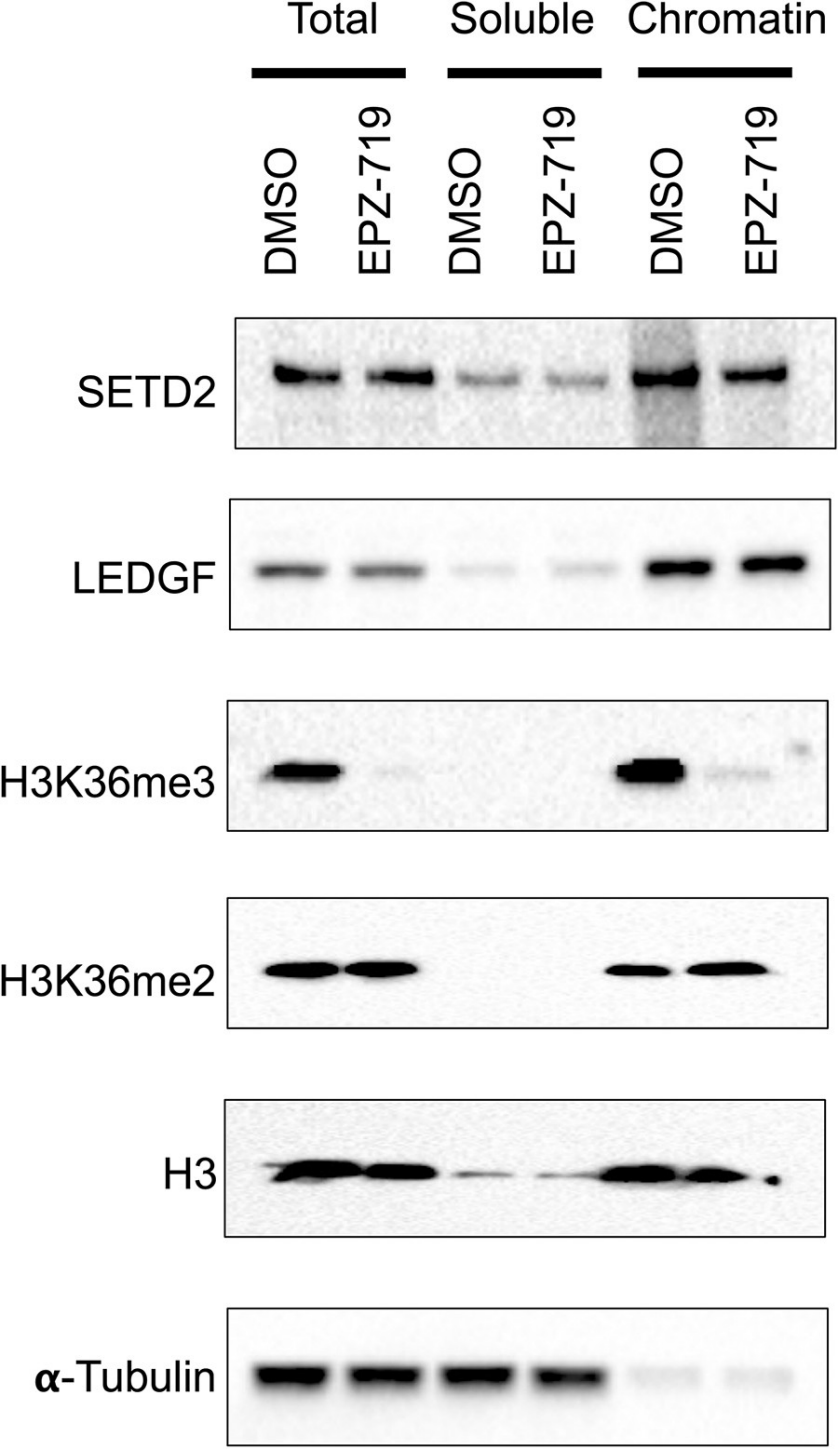
LEDGF associates with cellular chromatin in a H3K36me3-independent manner. 2D10 cells were exposed to EPZ-719 at 500nM or control vehicle (DMSO) for three days, then fractionated into total cellular lysate, soluble lysate and chromatin-associated proteins. Protein samples were then analyzed by western blot for the indicated targets.

H3K36me3 modified histones mediate HIV integration in vitro [53], and HIV integration sites in vivo are enriched in H3K36me3 abundant regions [54]. Nevertheless, our data indicate that HIV integration is independent of H3K36me3. The HIV pre-integration complex has been shown to interact with chromatin though the H3K36me3 binder LEDGF [55]. We therefore hypothesized that in the absence of H3K36me3, LEDGF-dependent integration could still occur through an alternative binding partner for LEDGF. For example, LEDGF has been shown to interact with H3K36me2 [56]. To explore this idea, we examined the association of LEDGF with cellular chromatin in the presence or absence of H3K36me3. We exposed 2D10 cells to EPZ-719 for 3 days, then fractionated the cells into soluble and chromatin-associated protein fractions, before western blotting for LEDGF as well as H3K36me3, H3K36me2, SETD2, total histone H3 and alpha-tubulin. As expected, H3K36me3 and H3K36me2 were both found predominantly in the chromatin fraction, while alpha tubulin was found primarily in the cytoplasm (**Fig 9**). Consistent with our previous observations, EPZ-719 caused dramatic depletion of H3K36me3 in total cellular lysates and within the chromatin-associated protein. Notably, both LEDGF and H3K36me2 remained abundantly associated with chromatin in the presence of EPZ-719. These results indicate that the association of LEDGF with chromatin is not dependent on H3K36me3, and that another mechanism, such as possibly H3K36me2, mediates chromatin binding by LEDGF in the absence of H3K36me3.

### H3K36me3 loss enhances sensitivity of latent HIV to reactivation with an HDAC inhibitor

**Fig 10:**
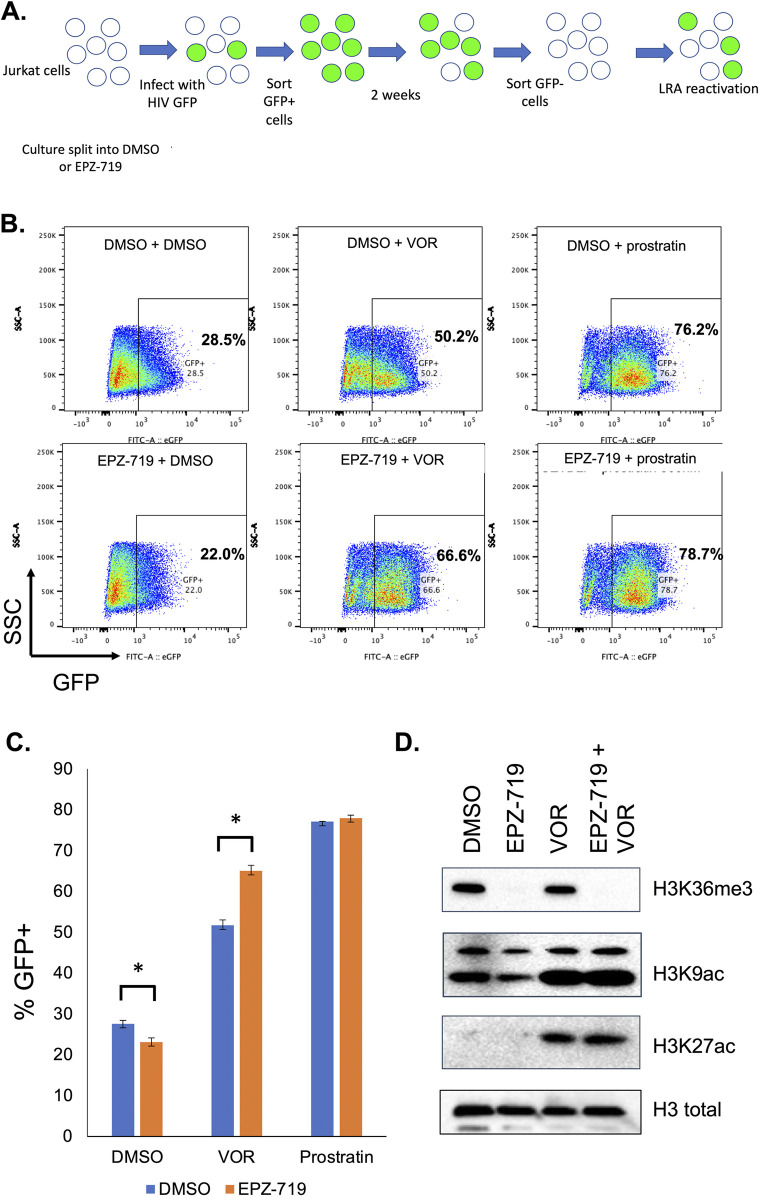
H3K36me3 depletion enhances sensitivity of latent HIV to reactivation with an HDAC inhibitor. (**A**). Schematic overview of experimental design. Jurkat cells were exposed to EPZ-719 (500nM) or DMSO for three days, then infected with HIV-dreGFP. At 48hpi, productively infected cells were isolated by flow sorting, then cultured in EPZ-719 (500nM) or DMSO for an additional two weeks. Latently infected cells (GFP-) were then enriched by flow sorting and stimulated with vorinostat (VOR) (500nM) or prostratin (500nM) in the presence of EPZ-719 (500nM) or DMSO. 24h after stimulation, the reactivation of the latently infected population was measured by flow cytometry. (**B, C**). Flow cytometry and bar chart of HIV-dreGFP reactivation in response to vorinostat (VOR) or prostratin. (**D**). Protein extracts from stimulated and control cells were isolated and histone acetylation (H3K9ac and H3K27ac) as well as H3K36me3 were measured for each population by western blot. Each datapoint represents the average of triplicate samples. Error bars represent the standard deviation of the mean. Asterisk represents a comparison where P<0.05 (T test).

The presence of latent proviruses is a key barrier to cure for HIV, but reactivation of latent proviruses is typically inefficient. We next decided to examine whether depletion of H3K36me3 from infected cells affects the reactivation of latent HIV using latency reversing agents (LRAs). To test this hypothesis, we exposed Jurkat cells to EPZ-719 for 3 days to deplete H3K36me3, then infected the cells with HIV-dreGFP. Actively infected cells were then enriched by flow sorting for GFP+ cells at 2dpi, then the infected cells were cultured for in continuous DMSO or EPZ-719 for an additional 20 days. After this period of culture, a latent (GFP-) population emerged, and these latently infected cells were further enriched by flow sorting for GFP- cells. Thus, we obtained a polyclonal population of Jurkat cells that were enriched for latently infected cells (**Fig 10A**). Nevertheless, in this population, a subset of cells (28%) still exhibited low level background HIV expression (**Fig 10B**). In the absence of stimulation, the EPZ-719 exposed cells exhibited a modestly lower level of baseline HIV expression (22% GFP+). We then stimulated the latently infected population with two LRAs with distinct mechanisms of action: vorinostat–a class 1 histone deacetylase (HDAC) inhibitor, and prostratin–a protein kinase C agonist. As expected, at 24h post stimulation, both LRAs reactivated a fraction of the latently infected cells, as indicated by an increase in the percent GFP+ cells (**Fig 10B and 10C**), with an average increase of ~22% GFP+ for vorinostat and an increase of ~48% for prostratin. Interestingly, while the response to prostratin was similar for the DMSO exposed cells and the EPZ-719 exposed cells, the EPZ-719 exposed cells exhibited a significantly stronger reactivation in response to vorinostat (increase of 44% GFP+ for EPZ-719 exposed cells vs 22% for DMSO exposed cells). To examine further whether this increased viral response to vorinostat in the absence of H3K36me3, was related to a global elevated responsiveness to vorinostat in terms of histone acetylation, we measured total H3K9 and H3K27 acetylation levels for the infected cell population in each of the conditions by western blot (**Fig 10D**). As expected, global H3K9ac and H3K27ac increased significantly in response to vorinostat exposure. Notably, this increase was similar for both EPZ-719 and DMSO exposed cells, suggesting that EPZ-719 does not impact global responsiveness to class 1 HDAC inhibitors, but instead may impact a specific set of genes including HIV. In some experiments, we also observed a small reduction in H3K9ac levels in the presence of EPZ-719 alone, but this was not consistently observed. The basis for the impact of EPZ-719 on latency reversal by vorinostat but not prostratin is unclear but suggests that H3K36me3 regulates a defined set of transcription controlling steps that connect to some LRAs but not others.

**Fig 11:**
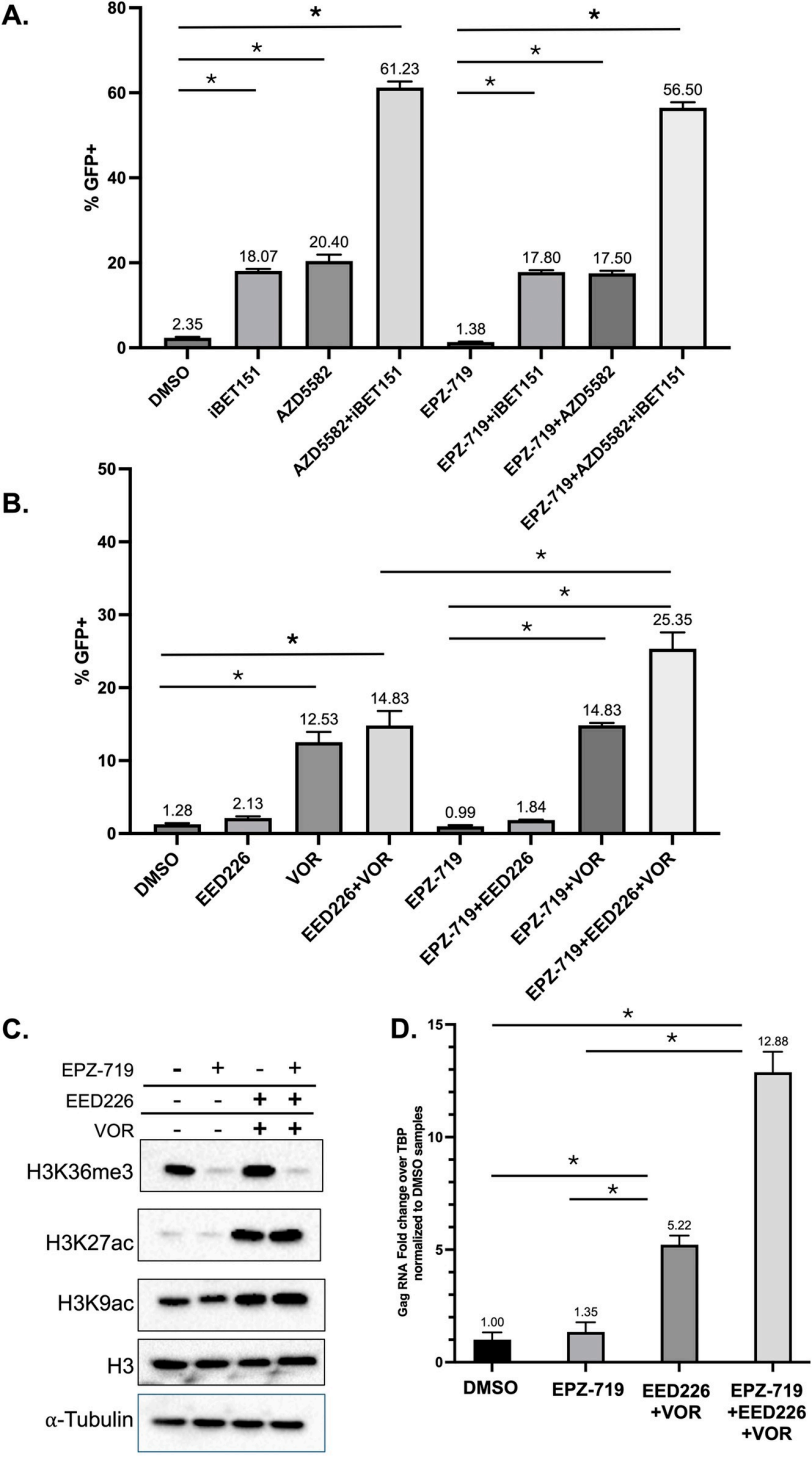
EPZ-719 enhances latency reversal by a vorinostat/EEDi combination in 2D10 cells. 2D10 cells were incubated with 500nM EPZ-719 for three days prior to stimulation for 24h with the small molecules indicated (EED226 = EED inhibitor, AZD5582 = non canonical NF-κB agonist, iBET151 = BRD4 inhibitor, VOR = vorinostat/HDAC inhibitor). Cells were then analyzed for reactivation of latent HIV by flow cytometry (**A, B**). (**C**) The abundance of cellular histone modifications was determined by western blot. (**D**). HIV Gag RNA was quantified by real-time PCR. Statistically significant differences are highlighted (P<0.05, one-way ANOVA).

We next examined the impact of H3K36me3 depletion in latency reversal in a different model of HIV latency. 2D10 cells contain a latent integrated copy of the HIV genome that encodes a destabilized GFP protein [57]. At baseline conditions, these cells do not express HIV gene products, but can be reactivated by a wide range of latency reversing agents. To determine how H3K36me3 depletion impacts responsiveness of these cells to LRAs, we exposed 2D10 cells to EPZ-719 or control vehicle (DMSO) alone, or in combination with different LRAs. As we have previously observed from de novo infection of Jurkat cells, EPZ-719 caused a small but reproducible drop in baseline viral expression of HIV and had no intrinsic LRA activity. When we combined EPZ-719 with the bromodomain inhibitor iBET151 or the non-canonical NF-κB agonist AZD5582, two LRAs that have been recently shown to synergistically reactivate latent HIV [58], we observed that EZP-719 had little effect on viral reactivation from each LRA alone or in combination (**Fig 11A**). In contrast, when we combined EPZ-719 with vorinostat and an inhibitor of the H3K27me3 binding protein EED (EED226), we observed significant enhancement of latency reversal by the vorinostat/EED226 combination (**Fig 11B**). Similar to our observations in a polyclonal population of HIV infected cells, the enhanced sensitivity of 2D10 cells to vorinostat-mediated reactivation in the presence of EPZ-719 was not associated with overall greater levels of histone acetylation after vorinostat exposure (**Fig 11C**). Since these observations rely on detection of viral protein expression by flow cytometry, we also examined whether the enhancement of vorinostat/EED226 mediated latency reversal in 2D10 cells could be observed at the level of viral RNA. When we examined expression of HIV Gag viral RNA (vRNA) by quantitative PCR, we observed that EPZ-719 strongly enhanced upregulation of vRNA in response to the vorinostat/EED226 combination, indicating that this effect is mediated at the transcriptional level (**Fig 11D**). Altogether these finding support the notion that SETD2/H3K36me3 levels in HIV infected cells can modulate the sensitivity of latent proviruses to some LRAs, particularly to vorinostat or to vorinostat/EEDi combinations.

## Discussion

Defining the set of host factors that regulate HIV expression will be essential for developing approaches to broadly reactivate the latent HIV reservoir. Studies have shown that HIV expression is regulated by an array of host cell transcriptional complexes and chromatin modifying enzymes [59,60]. Curiously, latent HIV proviruses retain features of transcriptionally repressed heterochromatic regions despite HIV integration occurring primarily in actively transcribed host genes. Since many of these chromatin features can be vertically transmitted during cell division, latency likely represents a stable and heritable epigenetic state. Thus, in addition to being important for direct repression of HIV expression within a cell, latency-associated chromatin likely plays a key role in reservoir persistence by transmitting the latent state during clonal expansion of infected cells in people with HIV [10,14,16,61]. Histone-modifying enzymes have been examined for specific roles in HIV latency and reactivation and various complexes have been found to play positive or negative roles in HIV expression. In particular, the role of histone deacetylases (HDACs) in the establishment and maintenance of latency is well known, and HDAC inhibitors reverse and prevent HIV latency, both in cell-based model systems and in PWH [18,22,37,62–64]. The histone methyltransferase EZH2, the catalytic subunit of the polycomb repressive complex 2 (PRC2), may also play a role in HIV silencing [11,65–67]. More recently, the HMTs SMYD2 and SMYD5 have been implicated in HIV expression and latency. SMYD5 has been shown to be required for high HIV expression and promotes HIV transcription by binding to the TAR RNA and mediating methylation of Tat [68,69].

Notably, the role of the SETD2 in HIV infection has not been previously examined. SETD2 is a large (288kDa) protein that is abundantly expressed in mammalian cells and is solely responsible for generating H3K36me3 [27,70]. The functional role of H3K36me3 in transcription and gene expression appears to be complex. H3K36me3 is highly enriched in the gene bodies of actively transcribed genes and has been shown to repress cryptic transcriptional initiation sites within genes through the recruitment of HDACs and other repressive modifications, including DNA methylation [28,71,72]. Other data suggests that H3K36me3 limits the spread of the repressive H3K27me3 mark within actively expressed genes [73] in addition to modulating post-transcriptional events that include RNA processing and m6A methylation of RNA [44]. Notably, SETD2 has been shown to play a role in viral replication. The SARS-Cov2 protein NSP9 inhibits the interferon response pathway through targeting SETD2 [74], and SETD2 has also been implicated in transcriptional regulation of human papilloma virus (HPV) [75,76]. As such, in this manuscript we have investigated the role of SETD2 in HIV gene expression and latency.

We found in this work that SETD2 expression and its activity is important for promoting high HIV expression in infected cells and for limiting the emergence of a latently infected cell population. By using a selective inhibitor of SETD2 or by CRISPR-mediated knockout of SETD2, we show that loss of SETD2 and H3K36me3 was associated with significantly reduced HIV expression and an increased fraction of cells with a latent viral phenotype. This finding was confirmed in both Jurkat cell and primary cell models of HIV infection. Notably H3K36me3 depletion did not have an impact on the overall number of infected cells after adding HIV virus particles to target cells, indicating that SETD2 and H3K36me3 are not required for HIV infection and integration but affect a post-integration aspect of viral gene expression. We observed that HIV integration sites were largely similar in the presence or absence of H3K36me3 in terms of chromosomal distribution and preference for highly transcribed genes. Nevertheless, we did observe a consistent shift in the integration sites towards non genes in the absence of H3K36me3, and a redistribution away from actively transcribed regions to quiescent and polycomb repressed chromatin regions. Given that the H3K36me2 mark generated by NSD enzymes is also predicted to recruit LEDGF, we speculate that H3K36me2 plays a compensatory role in the absence of SETD2/H3K36me3. Consistent with this hypothesis, LEDGF and H3K36me2 both remain associated with chromatin in the presence of a SETD2 inhibitor.

Based on our findings we propose that the effect of SETD2 on HIV expression is at least in part at the post-transcriptional level. Despite robust changes to HIV expression at the protein level in EPZ-719 exposed or SETD2-depleted cells, we observed no change to the overall level of HIV RNA in either RNAseq data or in qPCR for viral RNA. SETD2 has previously been shown to affect several aspects of post-transcriptional RNA processing. In particular, SETD2 was shown to be required for m6A methylation of cellular RNA, and this RNA modification is known to strongly impact gene expression. Other labs have also previously shown that the HIV RNA is extensively methylated with m6A [45–48]. Nevertheless, we did not observe any effect of SETD2 on total or HIV m6A RNA methylation in our study. It remains possible that SETD2 affects a different less well characterized form of RNA modification that is required for efficient HIV expression.

We also examined HIV RNA transcript splicing and found that SETD2 inhibition modestly affected the fraction of spliced transcripts. Specifically, overall HIV splicing was reduced in the presence of EPZ-719. Furthermore, within spliced transcripts some modest differences in donor/acceptor usage were also observed. These data suggest that the presence of SETD2 and H3K36me3 on HIV provirus-associated histones during transcription affects post transcriptional maturation of viral RNA. It is possible that the altered transcript splicing pattern for HIV in the absence of SETD2 activity contributes to the reduced overall level of viral gene expression. However, it remains possible that other post transcriptional mechanism also contribute to SETD2’s role in HIV expression.

It is also possible that SETD2 exerts an indirect effect on HIV expression. Indeed, global transcriptomic profiling of EPZ-719 exposed cells indicated altered expression of many cellular genes. Interestingly, increased expression of several interferon sensitive genes (ISGs) was observed when SETD2 activity was inhibited, suggesting induction of an overall antiviral state that could contribute to inhibiting HIV expression. Notably, previous work has linked SETD2 to the innate immune system. Specifically, SETD2 has been shown to amplify interferon (IFN) signaling by methylating STAT1 [77]. It is important to note that SETD2 has been suggested to methylate cellular targets other than H3K36, which could conceivably be important for regulation of HIV. For example, in addition to STAT1, SETD2 has been suggested to trimethylate actin on K68 [78].

Interestingly, depletion of H3K36me3 also increased the sensitivity of latent proviruses to reactivation with the histone deacetylase inhibitor vorinostat. The mechanism behind this phenomenon is unclear, but previous work has shown that inhibiting the HMT EZH2 can also enhance the responsiveness of latent proviruses to vorinostat [66]. H3K36me3 represses transcriptional initiation within gene bodies, but it is also possible that in infected cells, H3K36me3 is also found at the viral LTR where it could contribute to suppression of HIV transcription. Indeed our experiments revealed abundant H3K36me3 across the proviral genome, including at the LTR. Although we found that SETD2 inhibition alone did not impact HIV transcription, removal of LTR-associated H3K36me3 could nevertheless render a subpopulation of viral promoters in a poised state that responds to HDAC inhibition, in addition to a post-transcriptional effect. Depleting H3K36me3 with EPZ-719 may also help to improve the responsiveness of the clinical reservoir to vorinostat. However, an obstacle to this approach is the apparently limited efficacy of EPZ-719 in resting CD4 T cells. This low activity likely results from slow turnover of H3K36me3 in resting CD4 T cells, and it is possible that enhancing demethylation of this residue could achieve a similar effect to inhibiting SETD2.

Finally, a key question raised by our findings is whether loss of SETD2 activity or H3K36me3 depletion is associated with the formation of maintenance of the clinical reservoir. Variation in SETD2 expression or activity and/or abundance of H3K36me3 could impact whether a given infected cell adopts a latent or active infection phenotype. Additional studies will be needed to demonstrate the role of SETD2 in regulation of the clinical reservoir and its potential as a target to enhance latency reversal in vivo.

## Methods and materials

### Cell lines

HEK293T cells were maintained in DMEM media with 10% fetal bovine serum (FBS) and penicillin/streptomycin. Jurkat cells, CEM5.25 cells and primary CD4 T cells were maintained in RPMI media with 10% FBS, penicillin/streptomycin, and supplemented with glutamine, HEPES and sodium pyruvate. 2D10 cells were a gift from Dr Jonathan Karn. CEM5.25 cells were provided by Dr Dan Littman. 293T cells were obtained from ATCC.

### Virus infections

HIV particles were produced by transfection of HEK293T cells with viral plasmid DNA using Mirus LT1 reagent (Mirus Bio LLC, Madison WI). At 24h, supernatant was replaced with fresh RPMI to collect virus. At 48h, viral supernatant was collected. Cellular debris was removed first by low-speed centrifugation (300g, 5min), then filtration through a 0.45μm filter. The clarified supernatant was then frozen in aliquots and titered by infecting Jurkat cells with a dilution series of virus supernatant followed by flow cytometry for GFP+ cells at 48h. For the HIV-dreGFP virus (NL4-3-D6-dreGFP/Thy1.2), viral backbone plasmid was co-transfected with two packaging plasmids–PAX2 (encoding HIV GagPol) and MD2-VSVG (encoding the VSV-G glycoprotein). NL4-3-D6-dreGFP/Thy1.2 was modified from a parental plasmid provided by Dr R Siliciano. The replication competent NL4-3 strain clone was obtained from Aidsreagent.org.

### Flow cytometry and cell sorting

Samples of infected cells were analyzed using a Fortessa flow cytometer (Becton Dickson). Viable cells were gated using a live/dead stain–Zombie Violet (Biolegend) to stain cells prior to analysis. Doublet cells were excluded using forward scatter and side scatter. For flow sorting to enrich cells based on GFP expression, a FACSAria machine (Becton Dickson) in BSL2+ containment facility was used.

### Primary cell model of HIV latency

Human whole blood was obtained from StemCell Inc (Vancouver), and total CD4 T cells isolated using a negative selection CD4 T cell enrichment kit (StemCell). Aliquots of CD4 T cells were frozen and stored in 90% FBS 10% dimethylsulfoxide (DMSO). Aliquots of frozen cells were then thawed and activated with anti-CD3/CD28 beads for 48h (Life Technologies). Activated CD4 T cells were then spinocculated with HIV-dreGFP virus supernatant for 2h at 600g, before plating in RPMI with IL-2 (100U/mL) and IL-7 (5ng/mL). Infection was measured at 48h post infection by flow cytometry. Cell media was replaced every 2–3 days, and the cells were maintained at a density of between 1–2 million per mL. In some experiments, GFP+ cells were sorted at 48 hours post infection to enrich for actively infected cells using a FACSAria (Becton Dickson).

### CRISPR-Cas9 gene knockouts

CRISPR RNA targeting sequences (crRNA) against SETD2 were designed using CRISPick [79], while Tat-targeting and non-targeting control sequences were derived from previous literature [80, 81]. CRISPR-Cas9 ribonucleoprotein complexes (RNPs) were prepared by mixing crRNA targeting sequence and tracrRNA (IDT) at 1:1 to a final concentration of 100μM and annealed in duplex buffer (IDT) by heating to 95°C and slow cooling to room temperature in a PCR thermocycler. RNP complexes were generated immediately before nucleofection by mixing 0.8μL of Cas9 enzyme (62μM concentration, 49.6pMol total, ALT-R by IDT), 0.8μL of poly-glutamic acid (15kDa average polymer size, 100mg/mL concentration, Alamanda laboratories), and 1μL of annealed duplex crRNA:tracrRNA (100pMol for a final molar ratio of approximately 2:1 RNA:Cas9 enzyme). For SETD2 targeting, three crRNAs were multiplexed for more efficient target knockout. Primary CD4 T cells were isolated, activated, and infected as above. Six days after activation (4 days post infection), 3x10^6^ cells per condition were pelleted at 90g for 10 minutes, washed in PBS, and then resuspended in primary cell nucleofection buffer P3 (Lonza) at 1x10^8^ cells per mL (2x10^6^ cells per 20μL reaction volume). Cell suspensions were then added to RNP complexes, and nucleofection was performed using the CM137 protocol (Lonza 4D nucleofector). Nucleofected cells were then immediately resuspended in pre-warmed RPMI and incubated at 37°C for 20 min prior to resuspension at 2x10^6^ cells per mL in complete RPMI with IL-2 and IL-7.

Specific crRNA sequences used were:

SETD2

AltR1/rGrG rArGrU rCrGrA rGrUrC rUrArC rCrUrG rArArG rGrUrU rUrUrA rGrArG rCrUrA rUrGrC rU/AltR2/

/AltR1/rGrC rUrCrA rArGrG rUrGrA rArArU rArGrC rArUrG rGrUrU rUrUrA rGrArG rCrUrA rUrGrC rU/AltR2/

/AltR1/rArU rGrArA rCrUrG rGrGrA rUrUrC rCrGrA rCrGrA rGrUrU rUrUrA rGrArG rCrUrA rUrGrC rU/AltR2/

Non-Targeting(NT) /AlTR1/rArCrGrGrArGrGrCrUrArArGrCrGrUrCrGrCrArArGrUrUrUrUrArGrArGrCrUrArUrGrCrU/AlTR2/

Tat

/AlTR1/rCrCrUrUrArGrGrCrArUrCrUrCrCrUrArUrGrGrCrGrUrUrUrUrArGrArGrCrUrArUrGrCrU/AlTR2/

### Cellular fractionation and western blotting

To produce protein lysates, cells were pelleted by centrifugation (300g 5min), then washed in phosphate buffered saline (PBS), before being lysed in RIPA buffer (ThermoFisher) supplemented with protease inhibitors (Roche). Cell protein lysates were quantified by Bradford assay (ThermoFisher), then separated by Tris-Glycine SDS-PAGE, and transferred to polyvinylidene difluoride (PVDF) or nitrocellulose membranes. Blots were blocked in 5% milk in Tris-Buffered Saline (TBS), then incubated overnight with primary antibodies diluted in TBS containing 5% bovine serum albumin (BSA) or 5% milk and washed with 0.1% Tween20 TBS (TBST). Membranes were then stained with secondary antibodies (1:10,000) conjugated to horseradish peroxidase for 1h at room temperature and washed three times in TBST, followed by detection by enhanced chemiluminescence (ThermoFisher).

### RNAseq

Total RNA was harvested from triplicate cultures of 2–3 million HIV-dreGFP infected Jurkat cells exposed to EPZ-719 (500nM) or control (DMSO) using a RNEasy kit (Qiagen). RNA quantity and quality were then analyzed by nanodrop and Tapestation (Agilent) analysis respectively. RNA integrity number (RIN) scores were between 9.9 and 10.0. RNAseq libraries were then prepared using the KAPA Total RNA library prep kit, followed by 50bp paired end sequencing on a Nextseq2000 P2 flow cell. 70–90 million reads were obtained per sample. To analyze the data, the raw reads were first filtered by FastQC and CutAdapt to remove low quality reads. RNAStar was then used to align the reads to Hg38 (patch 17), followed by featurecounts to quantify transcripts. Differentially expressed genes were then identified by DESeq2 [82].

### CUT&RUN

CUT&RUN was carried out as previously described [19], using a commercial kit from Epicypher. Infected Jurkat cells were analyzed in triplicate by washing and coupling to Concanavalin-A coated beads, before incubation overnight in a Digitonin (0.01%) containing buffer with targeting antibodies against H3K36me3, H3K4me3 or control IgG. The cells were then washed in permeabilization buffer and incubated with pAG-MNase for 15 minutes at room temperature. After additional washing, CaCl_2_ was added to initiate cleavage of antibody bound chromatin for 2h at 4°C. Released DNA was then eluted, combined with E Coli DNA spike-in, and barcoded libraries constructed using the Epicypher library preparation kit (Epicypher). Libraries were sequenced using an Illumina Novaseq sequencer. To analyze the data, fastq files were first trimmed to remove adapter sequences using CutAdapt, then aligned to a combined human (Hg38), E Coli and HIV reference genome using Bowtie2 [83]. Peaks with enriched regions of H3K36me3 were identified using MACS2 [84]. For visualization, bigwig files were scaled to normalize to the E Coli spike-in DNA.

### Quantitative PCR

HIV Gag unspliced RNA and Beta-actin RNA were quantified as previously described [40]. Briefly, cells were washed in PBS then lysed in RLTplus buffer (Qiagen) with 1% beta-mercaptoethanol. RNA was then isolated using an RNEasy plus kit (Qiagen) and eluted into nuclease free water. RNA samples were quantified by nanodrop, then 100ng of RNA was reverse transcribed and amplified using Fastvirus (Thermo, Waltham, MA) and primer sets for HIV Gag RNA (GAG-F: ATCAAGCAGCCATGCAAATGTT, GAG-R: CTGAAGGGTACTAGTAGTTCCTGCTATGTC, GAG-Probe: FAM/ZEN-ACCATCAATGAGGAAGCTGCAGAATGGGA-IBFQ) and Beta-actin (BAC-F: TCACCCACACTGTGCCCATCTACGA, BAC-R: CAGCGGAACCGCTCATTGCCAATGG, BAC-Probe: HEX-ATGCCCTCCCCCATGCCATCCTGCGT-IBFQ). The reaction plate was run on a QS3 (Applied Biosystems, Foster City, CA) real time thermocycler with a 5 minute reverse transcription step at 50°C, followed by 40 cycles of 94°C (3 sec.), 60°C (30 sec.).

### Nuclear run on

To label and isolate newly synthesized RNA transcripts, we used a recently described approach for nascent RNA capture [85] using a Click-iT nascent RNA capture kit (Thermo). Infected cells were incubated with the cell permeable uridine analog 5-ethynyluridine (EU) at 500μM for one hour, followed by lysis and RNA extraction using an RNEasy kit (Qiagen). Biotin was then conjugated to labeled RNA in vitro, then RNA was precipitated overnight with glycogen, ammonium acetate and ethanol. Biotinylated RNAs were then isolated from the overall sample using Streptavidin T1 Dynabeads (Life Technologies). Reverse transcription was then immediately carried out on the beads using Superscript VILO reverse transcription kit. cDNAs were eluted, and targets were quantified using qPCR as described above.

### HIV splicing quantification

Quantification of HIV-1 transcripts was done similarly as described in [49], but adapted to use a random 14 base cDNA primer coupled with Illumina/MiSeq platform sequences. Briefly, the random reverse primer serves as a unique molecular identifier, and also primes across all viral RNAs as well as all cellular RNAs. HIV-1 specific RNAs are polymerase chain reaction (PCR) amplified using a primer just upstream of the major HIV-1 splice donor D1, which also adds Illumina platform sequences, and a downstream primer complementary to a common sequence at the 5’ end of the random reverse primers. The resulting libraries were sequenced using Illumina paired-end 300 base reads. Sequencing data was analyzed using an in-house script (available from the Swanstrom lab, UNC Chapel Hill) that combines information from forward and reverse reads to identify and quantify transcripts that either remain unspliced at D1 or splice to specific acceptors. For reads of sufficient length, transcripts that are spliced or unspliced at D4 (completely or incompletely spliced) can also be quantified.

### Integration site analysis

Genomic DNA was isolated using the DNeasy Blood and Tissue kit (Qiagen) per manufacturers protocols. Integration sites were determined using a linear amplification mediated PCR protocol adapted from methodology originally described in [50]. Duplicate PCR reactions gDNA were subject to linear amplification using the biotinylated primer am948 (sequence listed below). Resulting linear amplification products were pooled and purified using the NucleoSpin Gel and PCR Purification kit (Takara) with modified protocols for ssDNA (1:2 NTI buffer dilution). All recovered material was subject to streptavidin immunoprecipitation using the Dynabeads kilobaseBINDER Kit (ThermoFisher) using 5μL beads per manufacturers protocol to recover the biotinylated linear amplification product, washed, and followed by second strand synthesis on bead using Klenow (New England Biolabs, NEB) as described [50]. Post all enzymatic reactions, DNA-bound beads were washed twice in a Tween wash buffer (5mM Tris pH8, 1M NaCl, 0.5mM EDTA, 0.05% Tween-20) and once in 10mM Tris pH8. Double stranded on-bead DNA was digested with a mix of three blunt cutting enzymes (SspI-HF, StuI, and HincII) (NEB) to generate blunt-end DNA. A blunt-ended double stranded adapter based of sequences from [50] was generated by annealing two oligos, a sense strand with 5’ unpaired region and antisense with a 3’ blocked end. The adapter was ligated on to digested DNA using the NEB Quick Ligation Kit. Material was subject to on-bead PCR using primers am954/956 using the Platinum SuperFi II Mastermix (ThermoFisher/Life Technologies) for 15 cycles. 2μL of the first round PCR was then subject to nested PCR using am950/955 for 30 cycles using SuperFi II. PCR products were cleaned up using the Takara Nucleospin kit per manufacturers protocol and concentration and size range assessed using the D5000 Screentape (Agilent). 200fmol of each sample was barcoded and subject to library prep using the Native Barcoding Ligation Kit with V14 chemistry (SQK-NBD-114-24) per manufactures instructions. 20fmol of the pooled, barcoded library was loaded onto an MIN114 flow cell and run on the Nanopore Mk1C. Resulting FAST5 or POD5 files were basecalled and demultiplexed using the super high accuracy model for Dorado (Oxford Nanopore). Custom adapters sequences ligated during the protocol were trimmed using Cutadapt and successful trimming confirmed using FastQC with a custom adapter list pre- and post-trimming. Resulting reads were initially aligned to the reference HIV genome using minimap2 and non-aligning reads discarded. Reads were then realigned to a hybrid HIV-hg38 genome using minimap2 with the -Y option set to map chimeric reads. Reads were filtered using SAMtools [86] based on mapping quality (—min-MQ 20), mismatch rate (-e ‘[NM]/length(seq) < 0.02’), and SA tag. Reads with multiple SA alignments and viral reads not aligning to the first 1–10 bases of the reference were also removed. Two control samples of uninfected Jurkat gDNA processed concurrently with experimental samples yielded no viral or chimeric reads at this step. Overlapping reads collapsed using BEDTools merge [87]. Read depth of collapsed features was merged into the BED file using SAMtools bedcov with -d 10 and -c options. A reference BED file was generated using the hg.knowncanonical track cross-referenced with the knowntoRefSeq track from the UCSC Genome Table Browser for hg38 and filtered for duplicate annotations. BEDTools intersect was used to annotate the hybrid aligned BED file and filtered to produce a list of genomic regions with a minimum depth of 10 nanopore reads. To annotate chromatin state, we compared our final annotated BED file to the core 15-state ChromHMM model generated by the Roadmaps Epigenomics Project for sample E043, defined as a CD4-positive, CD25-negative helper T-cell (https://egg2.wustl.edu/roadmap/web_portal/meta.html). The E043 lift-over hg38 file for the core 15-state model was downloaded and BEDtools intersect used to annotate integration site locations.

### RNA methylation analysis

To measure the total abundance of m6A RNA in cells, 300ng of total RNA was analyzed using an m6A RNA methylation quantification kit (Epigentek) according to the manufacturer’s protocol. This kit uses a colorimetric enzyme-linked immunosorbent assay (ELISA) to detect m6A. ELISA plates were read at 450nm using a SpectraMax M3 plate reader (Molecular Devices). Samples were run in triplicate and compared to a standard curve of m6A. Abundance was calculated as a percentage of total RNA by mass. To measure m6A modification of HIV RNA, an EpiQuik CUT&RUN m6A RNA Enrichment (MeRIP) Kit (Epigentek) was used with an input of 10μg of total RNA from each condition and an m6A specific antibody or control IgG. Eluted RNAs were then analyzed using a quantitative PCR assay for an m6A rich region of the HIV RNA within the Env/Rev coding sequence. qPCR was run using an Applied Biosystems QS3. Primer sets used were Forward: GCC CGA AGG AAT AGA AGA AGA A, Reverse: GAT CGT CCC AGA TAA GTG CTA AG, Probe: 56-FAM/TG GAG AGA G/ZEN/A GAC AGA GAC AGA TCC A/3IABkFQ.

## Supporting information

S1 Fig EPZ-719 reduces H3K36me3 levels in a human T cell line. (**A**). 2D10 cells were exposed to a range of EPZ-719 concentrations for 24h. Whole cell protein lysates were then extracted and examined by western blot for H3K36me3, SETD2 and total histone 3 (H3) and β-tubulin. (**B**). 2D10 cells were exposed to 500nM EPZ-719 and total cellular protein extracted at times indicated. Extracts were then western blotted for H3K36me3, H3K36me2 and total histone 3 (H3).
(TIFF)

S2 Fig EPZ-719 does not inhibit Jurkat cell growth. Jurkat cells were seeded in RPMI at 250,000 cells per mL and incubate in the presence of 500nM EPZ-719 or DMSO for 4 days. At times indicated, cell density was measured. Each datapoint represents the average of triplicate wells. Error bars represent the standard deviation of the mean.
(TIFF)

S3 Fig No effect of EPZ-719 on primary T cell activation markers. Primary CD4 T cells were activated using anti-CD3/CD28 beads for 72h, then cultured in the presence of EPZ-719 (500nM) or DMSO for eight days. Cells were then stained for the T cell activation markers CD38 and HLA-DR. (**A**). Bar chart shows average percent double positive cells (CD38+/HLA-DR+) for three biological replicates. Error bars represent the standard deviation of the mean. NS = not significant (P>0.05, T test). (**B**). Representative flow cytometry plots for each condition are shown.
(TIFF)

S4 Fig HIV-mapping RNAseq read distribution. HIV-mapping reads within RNAseq data from 500nM EPZ-719 or DMSO exposed HIV-dreGFP infected Jurkat cells (From Fig 7 dataset) were visualized using the Integrative Genomics Viewer (IGV).
(TIFF)

S5 Fig EPZ-719 does not affect nascent viral RNA levels in Jurkat cells or viral RNA levels in HIV infected primary CD4 T cells. (**A, B**) HIV-dreGFP infected Jurkat cells were exposed to EPZ-719 (500nM) or control vehicle (DMSO) for 8 days, then pulse labeled with 5-ethnyluridine (EU) for one hour. RNA was then extracted and EU-labeled RNA biotinylated in vitro, followed by enrichment of biotinylated RNAs using streptavidin-coated beads. The presence of Gag viral RNA in the initial RNA sample (**A**) and in the enriched nascent RNA pool (**B**) was then quantified by real-time PCR. Data represent the average of three biological replicates. (**C**) Primary CD4 T cells were activated using anti-CD3/CD28 beads for three days then infected with HIV-dreGFP. From 48hpi, infected cells were then cultured in the presence of EPZ-719 (500nM) or control vehicle (DMSO) for 7 days. RNA was extracted and Gag RNA quantified by real-time PCR. Bars represent the average of four biological replicates. Error bars represent the standard deviation of the mean. NS = not significant (Students T Test).
(TIFF)

S6 Fig EPZ-719 does not affect global or HIV-specific m6A modification of RNA. **(A).** Schematic overview of experimental design. EPZ-719 was used at 500nM. (**B).** The abundance of latently infected (GFP-) Jurkat cells in the culture after 13 days of drug exposure was measured by flow cytometry. (**C).** The abundance of m6A RNA within the total RNA from DMSO or EPZ-719 exposed cells was measured by plate-based enzyme linked immunosorbent assay (ELISA). (**D).** m6A modification of HIV RNA was examined. Cellular RNA was immunoprecipitated with an m6A-specific antibody or control IgG followed by quantitative RT-PCR for a region of HIV located within the Env/Rev region. Each bar represents the average of biological triplicates. Error bars represent the standard deviation of the mean. NS = not significant, P>0.05 T Test.
(TIFF)

S7 Fig EPZ-719 affects HIV splicing in infected primary CD4 T cells. Primary CD4 T cells were activated with anti-CD3/CD28 beads, then infected with HIV-drEGFP for 2 days. The cells were then cultured in EPZ-719 (500nM) or DMSO for an additional eight days before RNA was harvested. HIV transcript splice variants were then quantified using the same method described in Fig 7. (**A**). The overall percentage of HIV transcripts that are spliced is shown for each condition. (**B**). The fraction of spliced viral transcripts that are fully spliced is shown for each condition. (**C**). Fraction of spliced viral RNAs using different initial viral splice acceptor sites (A1-A5) is shown for each condition. (**D**). Fraction of spliced viral RNAs using different final splice acceptor sites is shown for each condition. Each bar represents the average of four biological replicates. Error bars represent the standard deviation of the mean. Significant differences are highlighted (T Test). NS = Not significant (P>0.05).
(TIFF)

S8 Fig EPZ-719 affects splicing of cellular transcripts. RNAseq data from 500nM EPZ-719 or control (DMSO) exposed HIV infected cells at 8dpi was analyzed for differential splicing using DEXseq [88]. Genes with significantly different exon usage are highlighted in red (pval_adj_<0.05).
(TIFF)

S9 Fig Analysis of differentially expressed genes within the integration site gene set. (**A**) The fraction of differentially expressed genes (DEGs–Log_2_fold change>0.01, pval_adj_<0.05) within the total set of annotated human genes, and the set of genes with HIV integration sites in control (DMSO) or EPZ-719 exposed cells are displayed. (**B**) The fold change for all integration site genes that were also DEGs are shown for both control (DMSO) and EPZ-719 exposed conditions. Mean values for each condition shown as a horizontal bar.
(TIFF)

S1 Table DIfferentially expressed genes after EPZ-719 exposure. (XLSX)

S2 Table Enrichr analysis of upregulated DEGs. (XLSX)

S3 Table Enrichr analysis of downregulated DEGs. (XLSX)

S4 Table GO analysis of upregulated genes–biological process. (XLSX)

S5 Table GO analysis of upregulated genes–molecular function. (XLSX)

S6 Table GO analysis of downregulated genes–biological process. (XLSX)

S7 Table GO analysis of downregulated genes–molecular function. (XLSX)

S8 Table DEXseq analysis. (XLSX)
