# Supplementary figures and images for: The histone methyltransferase SETD2 regulates HIV expression and latency
Source: PLoS Pathog. 2024 Jun 7;20(6):e1012281. doi: 10.1371/journal.ppat.1012281 (PMC11189200; doi:10.1371/journal.ppat.1012281)

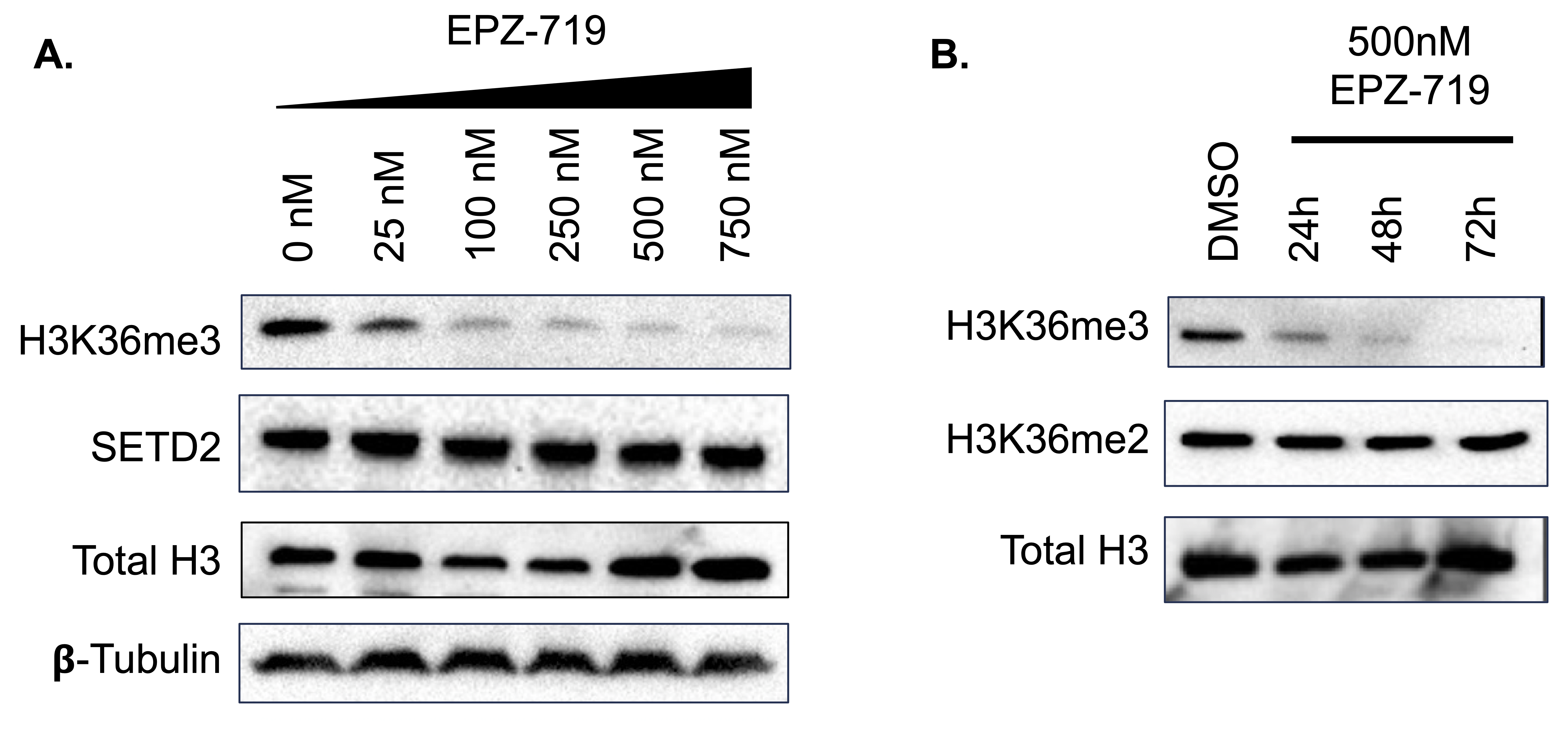

Supplement: S1 Fig — (A). 2D10 cells were exposed to a range of EPZ-719 concentrations for 24h. Whole cell protein lysates were then extracted and examined by western blot for H3K36me3, SETD2 and total histone 3 (H3) and β-tubulin. (B). 2D10 cells were exposed to 500nM EPZ-719 and total cellular protein extracted at times indicated. Extracts were then western blotted for H3K36me3, H3K36me2 and total histone 3 (H3). (TIFF) [file ppat.1012281.s001.tiff]

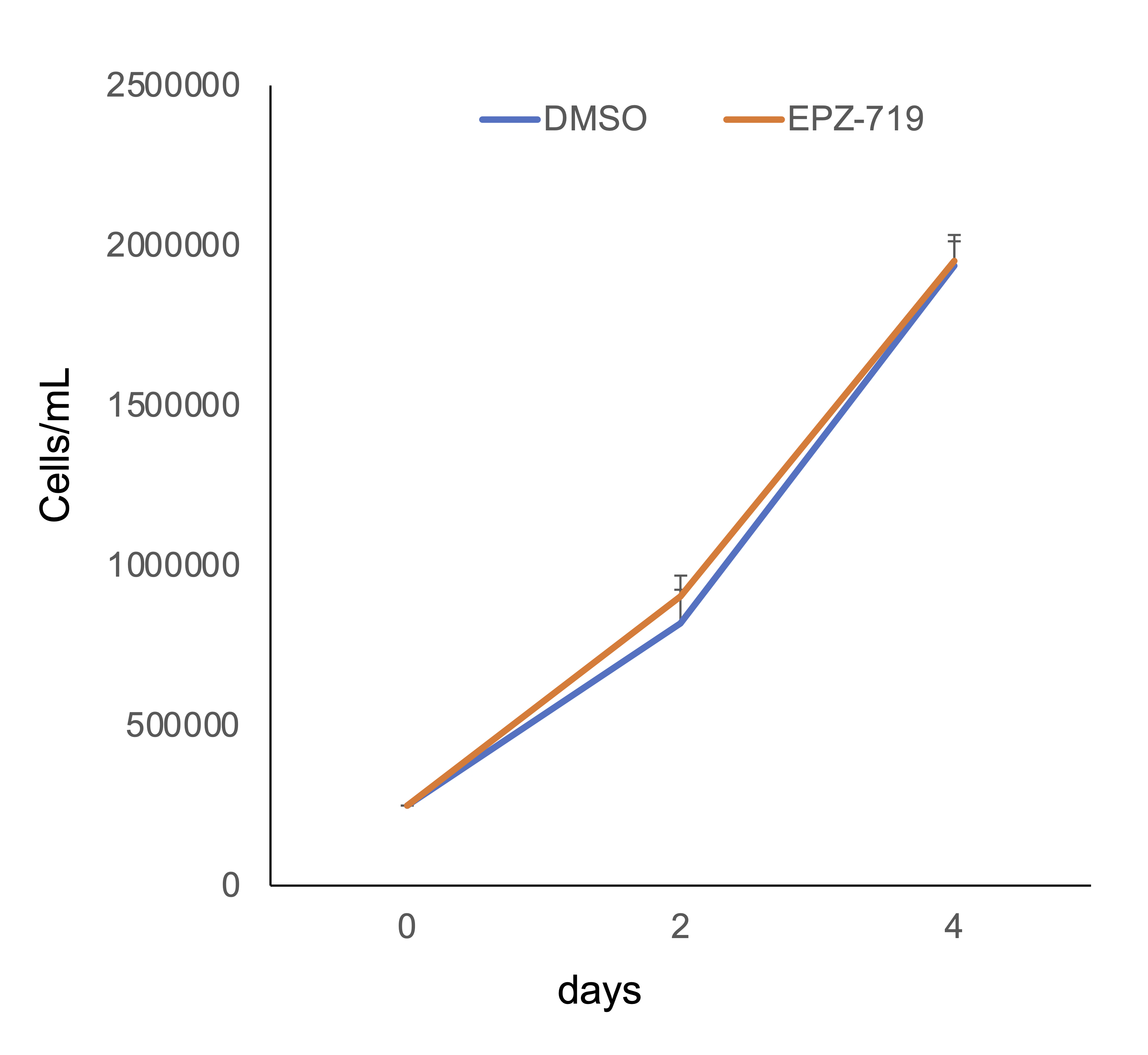

Supplement: S2 Fig — Jurkat cells were seeded in RPMI at 250,000 cells per mL and incubate in the presence of 500nM EPZ-719 or DMSO for 4 days. At times indicated, cell density was measured. Each datapoint represents the average of triplicate wells. Error bars represent the standard deviation of the mean. (TIFF) [file ppat.1012281.s002.tiff]

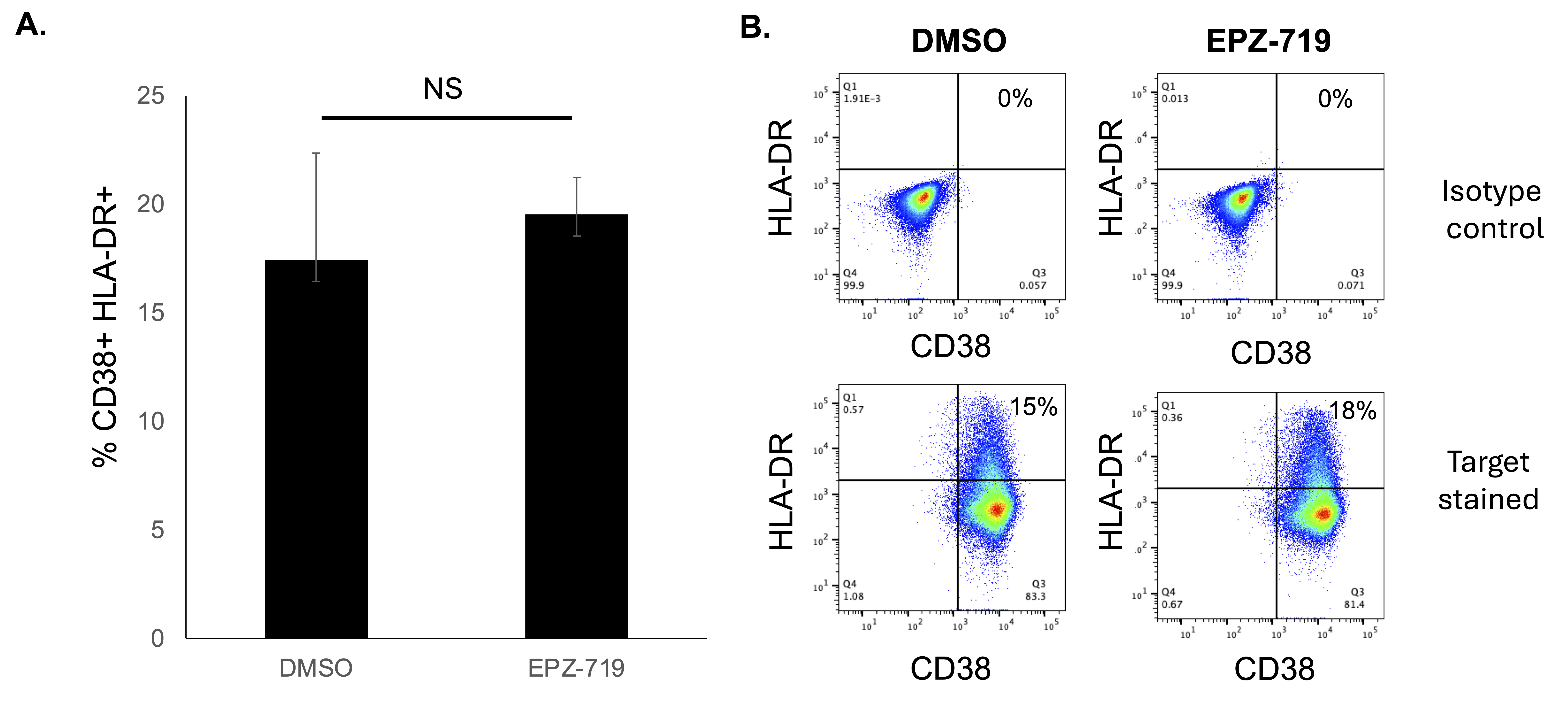

Supplement: S3 Fig — Primary CD4 T cells were activated using anti-CD3/CD28 beads for 72h, then cultured in the presence of EPZ-719 (500nM) or DMSO for eight days. Cells were then stained for the T cell activation markers CD38 and HLA-DR. (A). Bar chart shows average percent double positive cells (CD38+/HLA-DR+) for three biological replicates. Error bars represent the standard deviation of the mean. NS = not significant (P>0.05, T test). (B). Representative flow cytometry plots for each condition are shown. (TIFF) [file ppat.1012281.s003.tiff]

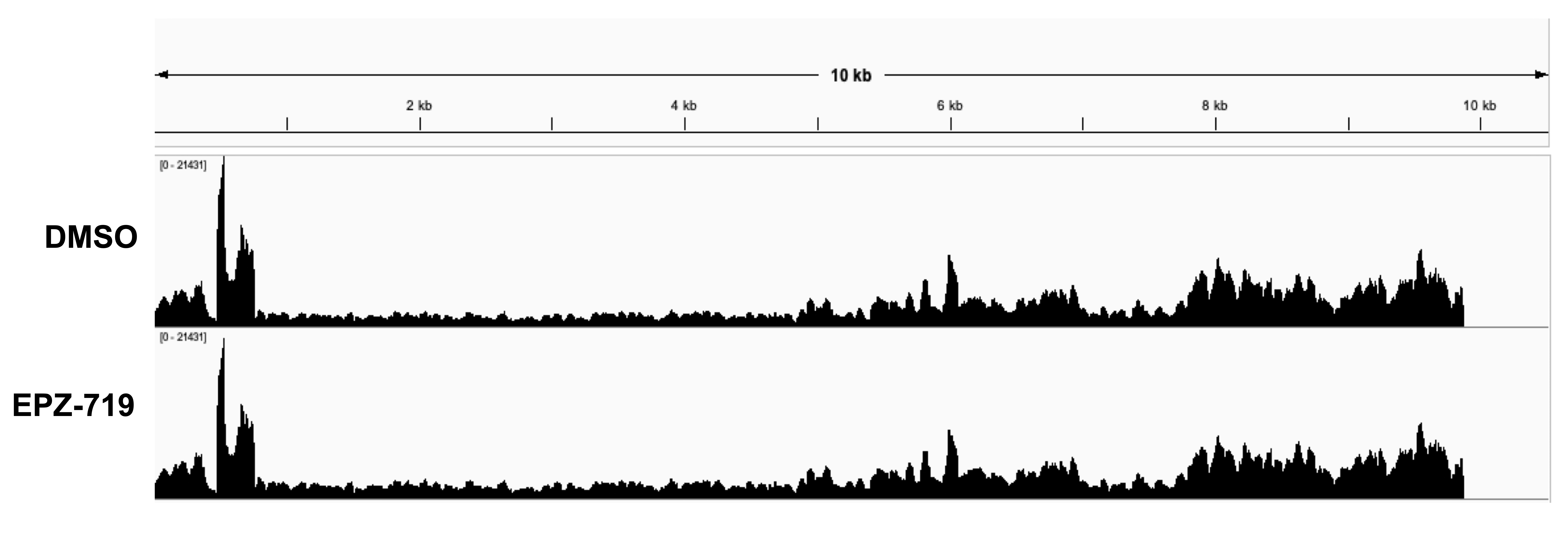

Supplement: S4 Fig — HIV-mapping reads within RNAseq data from 500nM EPZ-719 or DMSO exposed HIV-dreGFP infected Jurkat cells (From Fig 7 dataset) were visualized using the Integrative Genomics Viewer (IGV). (TIFF) [file ppat.1012281.s004.tiff]

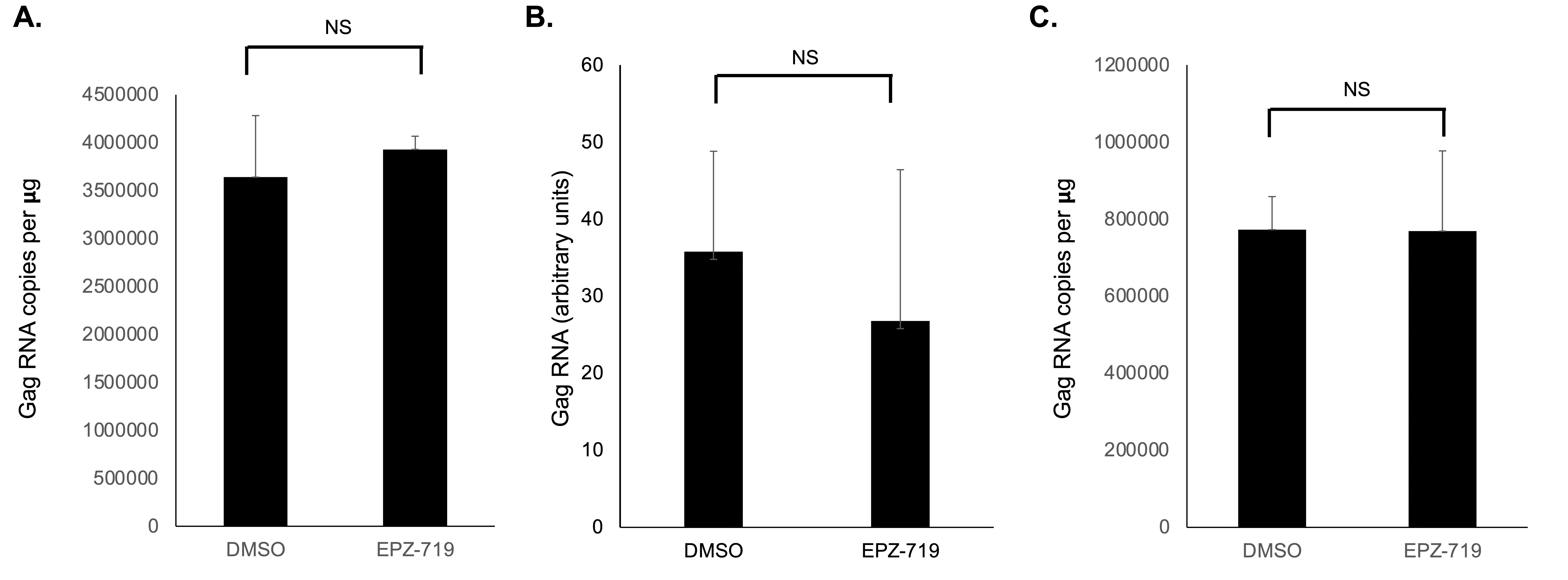

Supplement: S5 Fig — (A, B) HIV-dreGFP infected Jurkat cells were exposed to EPZ-719 (500nM) or control vehicle (DMSO) for 8 days, then pulse labeled with 5-ethnyluridine (EU) for one hour. RNA was then extracted and EU-labeled RNA biotinylated in vitro, followed by enrichment of biotinylated RNAs using streptavidin-coated beads. The presence of Gag viral RNA in the initial RNA sample (A) and in the enriched nascent RNA pool (B) was then quantified by real-time PCR. Data represent the average of three biological replicates. (C) Primary CD4 T cells were activated using anti-CD3/CD28 beads for three days then infected with HIV-dreGFP. From 48hpi, infected cells were then cultured in the presence of EPZ-719 (500nM) or control vehicle (DMSO) for 7 days. RNA was extracted and Gag RNA quantified by real-time PCR. Bars represent the average of four biological replicates. Error bars represent the standard deviation of the mean. NS = not significant (Students T Test). (TIFF) [file ppat.1012281.s005.tiff]

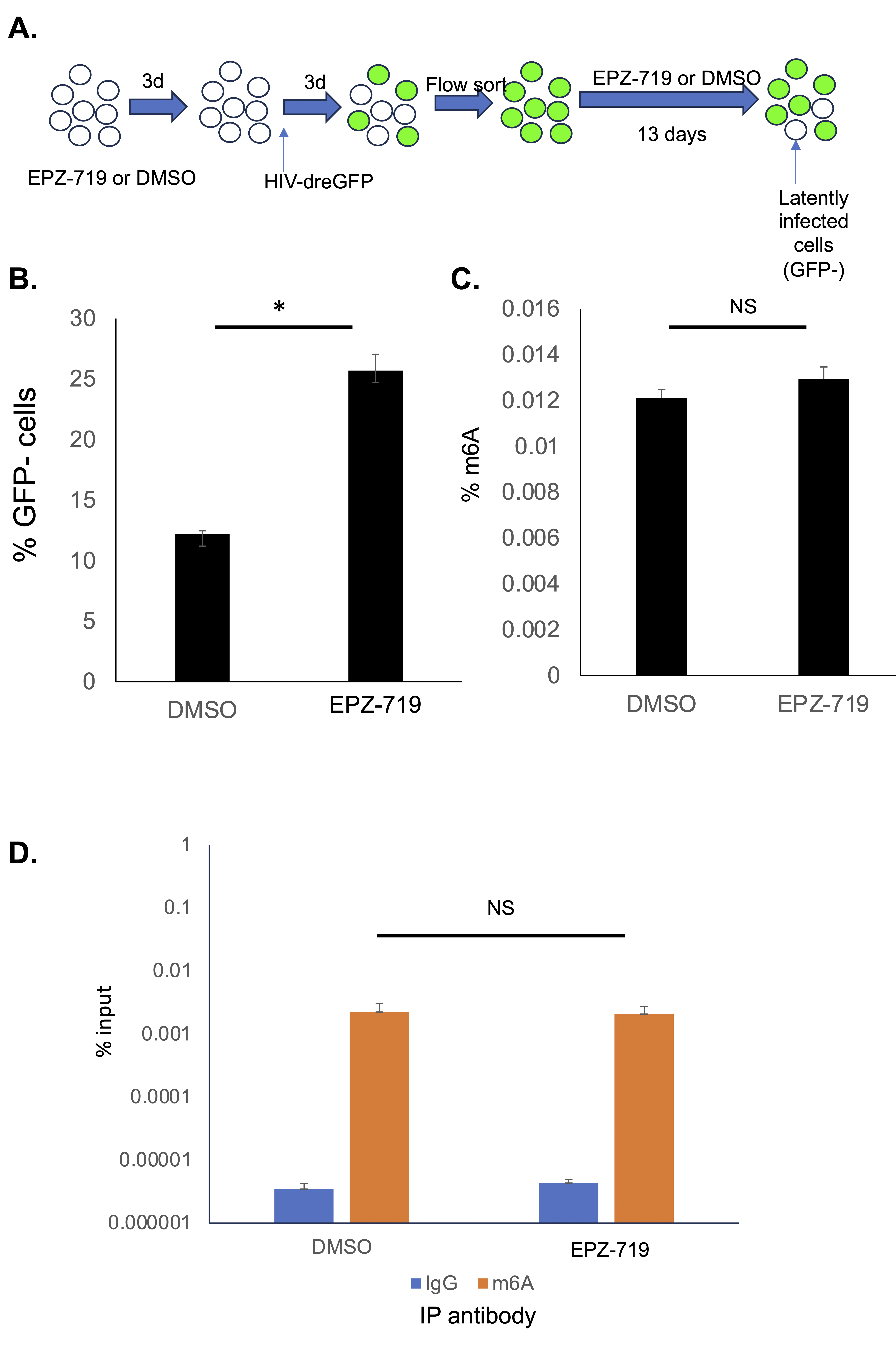

Supplement: S6 Fig — (A). Schematic overview of experimental design. EPZ-719 was used at 500nM. (B). The abundance of latently infected (GFP-) Jurkat cells in the culture after 13 days of drug exposure was measured by flow cytometry. (C). The abundance of m6A RNA within the total RNA from DMSO or EPZ-719 exposed cells was measured by plate-based enzyme linked immunosorbent assay (ELISA). (D). m6A modification of HIV RNA was examined. Cellular RNA was immunoprecipitated with an m6A-specific antibody or control IgG followed by quantitative RT-PCR for a region of HIV located within the Env/Rev region. Each bar represents the average of biological triplicates. Error bars represent the standard deviation of the mean. NS = not significant, P>0.05 T Test. (TIFF) [file ppat.1012281.s006.tiff]

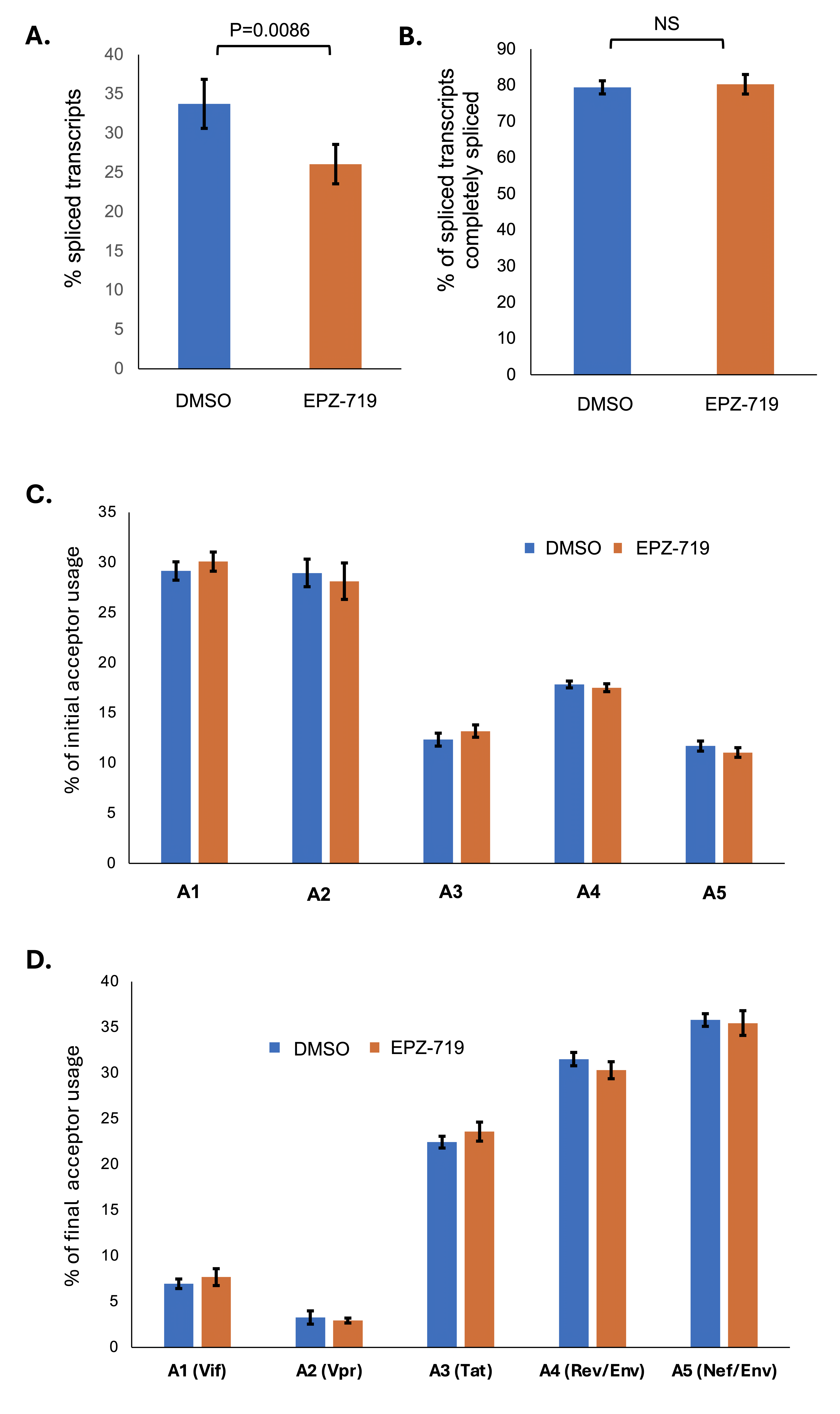

Supplement: S7 Fig — Primary CD4 T cells were activated with anti-CD3/CD28 beads, then infected with HIV-drEGFP for 2 days. The cells were then cultured in EPZ-719 (500nM) or DMSO for an additional eight days before RNA was harvested. HIV transcript splice variants were then quantified using the same method described in Fig 7. (A). The overall percentage of HIV transcripts that are spliced is shown for each condition. (B). The fraction of spliced viral transcripts that are fully spliced is shown for each condition. (C). Fraction of spliced viral RNAs using different initial viral splice acceptor sites (A1-A5) is shown for each condition. (D). Fraction of spliced viral RNAs using different final splice acceptor sites is shown for each condition. Each bar represents the average of four biological replicates. Error bars represent the standard deviation of the mean. Significant differences are highlighted (T Test). NS = Not significant (P>0.05). (TIFF) [file ppat.1012281.s007.tiff]

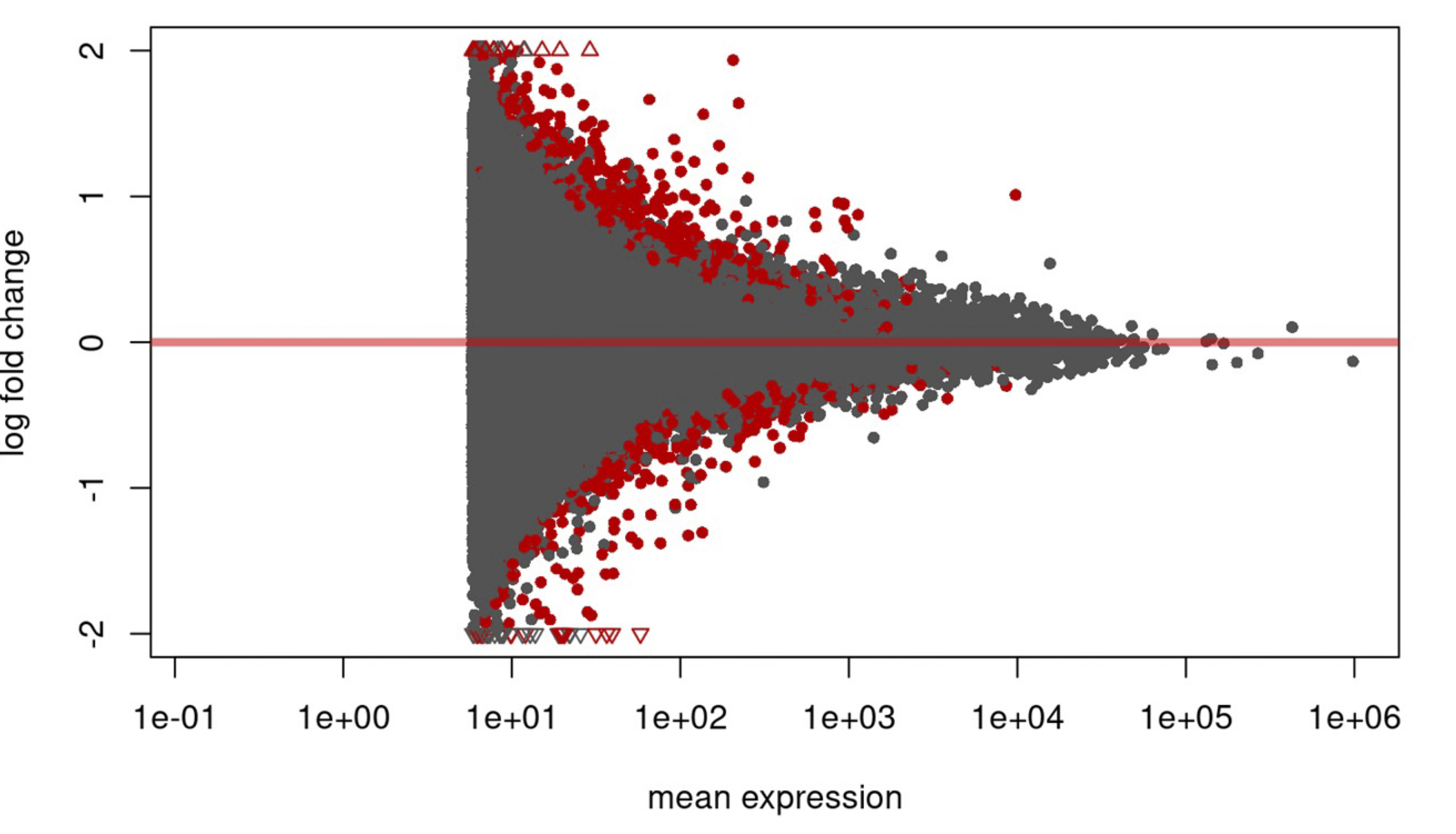

Supplement: S8 Fig — RNAseq data from 500nM EPZ-719 or control (DMSO) exposed HIV infected cells at 8dpi was analyzed for differential splicing using DEXseq [88]. Genes with significantly different exon usage are highlighted in red (pvaladj<0.05). (TIFF) [file ppat.1012281.s008.tiff]

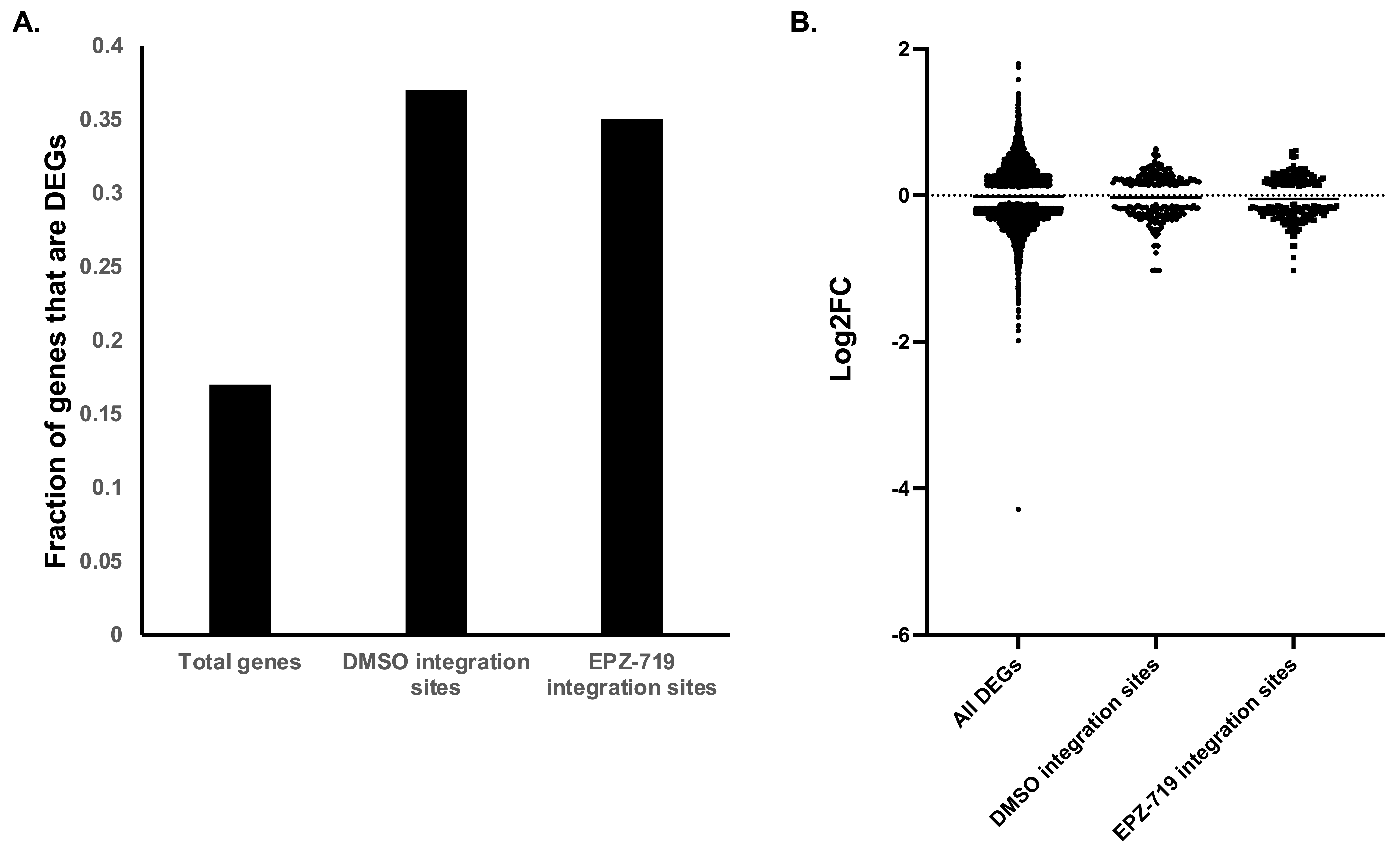

Supplement: S9 Fig — (A) The fraction of differentially expressed genes (DEGs–Log2fold change>0.01, pvaladj<0.05) within the total set of annotated human genes, and the set of genes with HIV integration sites in control (DMSO) or EPZ-719 exposed cells are displayed. (B) The fold change for all integration site genes that were also DEGs are shown for both control (DMSO) and EPZ-719 exposed conditions. Mean values for each condition shown as a horizontal bar. (TIFF) [file ppat.1012281.s009.tiff]
